# A census-based estimate of Earth's bacterial and archaeal diversity

**DOI:** 10.1371/journal.pbio.3000106

**Published:** 2019-02-04

**Authors:** Stilianos Louca, Florent Mazel, Michael Doebeli, Laura Wegener Parfrey

**Affiliations:** 1 Department of Biology, University of Oregon, Eugene, Oregon, United States of America; 2 Institute of Ecology and Evolution, University of Oregon, Eugene, Oregon, United States of America; 3 Biodiversity Research Centre, University of British Columbia, Vancouver, Canada; 4 Department of Zoology, University of British Columbia, Vancouver, Canada; 5 Department of Botany, University of British Columbia, Vancouver, Canada; 6 Department of Mathematics, University of British Columbia, Vancouver, Canada; Pacific Northwest National Laboratory, UNITED STATES

## Abstract

The global diversity of Bacteria and Archaea, the most ancient and most widespread forms of life on Earth, is a subject of intense controversy. This controversy stems largely from the fact that existing estimates are entirely based on theoretical models or extrapolations from small and biased data sets. Here, in an attempt to census the bulk of Earth's bacterial and archaeal ("prokaryotic") clades and to estimate their overall global richness, we analyzed over 1.7 billion 16S ribosomal RNA amplicon sequences in the V4 hypervariable region obtained from 492 studies worldwide, covering a multitude of environments and using multiple alternative primers. From this data set, we recovered 739,880 prokaryotic operational taxonomic units (OTUs, 16S-V4 gene clusters at 97% similarity), a commonly used measure of microbial richness. Using several statistical approaches, we estimate that there exist globally about 0.8–1.6 million prokaryotic OTUs, of which we recovered somewhere between 47%–96%, representing >99.98% of prokaryotic cells. Consistent with this conclusion, our data set independently "recaptured" 91%–93% of 16S sequences from multiple previous global surveys, including PCR-independent metagenomic surveys. The distribution of relative OTU abundances is consistent with a log-normal model commonly observed in larger organisms; the total number of OTUs predicted by this model is also consistent with our global richness estimates. By combining our estimates with the ratio of full-length versus partial-length (V4) sequence diversity in the SILVA sequence database, we further estimate that there exist about 2.2–4.3 million full-length OTUs worldwide. When restricting our analysis to the Americas, while controlling for the number of studies, we obtain similar richness estimates as for the global data set, suggesting that most OTUs are globally distributed. Qualitatively similar results are also obtained for other 16S similarity thresholds (90%, 95%, and 99%). Our estimates constrain the extent of a poorly quantified rare microbial biosphere and refute recent predictions that there exist trillions of prokaryotic OTUs.

## Abstract 2

A massive survey of Earth's Bacteria and Archaea reveals that their diversity is orders of magnitude lower than previously thought. The study also indicates that extinctions played an important role in prokaryotic evolution.

## Author summary

The global diversity of Bacteria and Archaea ("prokaryotes"), the most ancient and most widespread forms of life on Earth, is subject to high uncertainty. Here, to estimate the global diversity of prokaryotes, we analyzed a large number of 16S ribosomal RNA gene sequences, found in all prokaryotes and commonly used to catalogue prokaryotic diversity. Sequences were obtained from a multitude of environments across thousands of geographic locations worldwide. From this data set, we recovered 739,880 prokaryotic operational taxonomic units (OTUs), i.e., 16S gene clusters sharing 97% similarity, roughly corresponding to prokaryotic species. Using several statistical approaches and through comparison with existing databases and previous independent surveys, we estimate that there exist globally between 0.8 and 1.6 million prokaryotic OTUs. When restricting our analysis to the Americas, while controlling for the number of studies, we obtain similar estimates as for the global data set, suggesting that most OTUs are not restricted to a single continent but are instead globally distributed. Our estimates constrain the extent of a commonly hypothesized but poorly quantified rare prokaryotic biosphere and refute recent predictions that there exists trillions of prokaryotic OTUs. Our findings also indicate that, contrary to common speculation, extinctions may strongly influence global prokaryotic diversity.

## Introduction

Microorganisms are the most ancient and the most widespread form of life on Earth, inhabiting virtually every ecosystem and driving the bulk of global biogeochemical cycles. Culture-independent methods such as amplicon sequencing of 16S ribosomal RNA genes revealed the existence of a potentially vast undescribed microbial diversity, the full extent of which, however, remains highly controversial [1–9]. Determining the extent of this diversity remains an important but challenging task in our overall understanding of life, with major implications for ecological and evolutionary theory, environmental sciences and industry. Notably, a global census of microbial phylogenetic diversity, or at least knowledge of its full extent, is essential for reconstructing microbial evolution over geological time [10]. Estimates of global microbial diversity are also needed for scrutinizing proposed biodiversity scaling laws and macroecological theories [2,6,11]. Finally, undiscovered microorganisms may exhibit a large breadth of metabolic capabilities of particular interest to industry and medicine. An efficient exploration of this potential and realistic assessment of the feasibility of such an endeavor requires knowledge of the gaps in existing diversity databases [12–14].

The extent of global microbial diversity remains subject to intense controversy and widely diverging speculations [1–9]. This controversy stems largely from the fact that existing estimates are either based on extrapolations of empirical scaling laws [6], on theoretical biodiversity models [2], on data sets covering only a small fraction of global diversity [1,9], or on taxonomically biased databases, including mostly organisms that have been cultured or are of particular medical/industrial interest [3–5]. For example, Mora and colleagues [3] used the subset of currently named prokaryotic species to estimate that there exist approximately 10,000 bacterial species worldwide; this is clearly a strong underestimate, given that the SILVA sequence database [14] alone now contains hundreds of thousands of bacterial operational taxonomic units (OTUs), i.e., clusters of the 16S gene at 97% similarity—a traditional microbial species analog. Yarza and colleagues [4] and Schloss and colleagues [5] estimated that there exist a few million bacterial and archaeal ("prokaryotic") OTUs based on sequence discovery statistics in SILVA; however, environmental and taxonomic biases in SILVA [15] compromise the reliability of these estimates [16]. Larsen and colleagues [9] estimated that there exist billions of host-associated bacterial OTUs based on a heuristic and mathematically flawed extrapolation of bacterial OTU counts in typical insect species to all animal species (see the "Implications" section below for a detailed discussion). Locey and colleagues [6] even predicted that there exist trillions of microbial OTUs (at 97% similarity) based on an extrapolation of empirical scaling laws of local diversity in individual communities to global scales. Locey's estimate has fueled discussions about a potentially immense undiscovered microbial diversity and its uncertain ecological roles [16–20]. Locey's extrapolation of empirical scaling laws from local to global scales and across several orders of magnitude has been criticized and remains controversial [8,21].

Here, to address the above shortcomings, we attempted to explicitly census a large fraction of extant prokaryotic clades and used our census to estimate and chart total global prokaryotic OTU richness. For this census, we compiled massive publicly available raw Illumina 16S amplicon sequencing data from 34,368 samples across 492 studies, covering a wide range of environments from over 2,800 distinct geographical locations worldwide (S1 Fig). Environments covered include the surface and deep ocean, oxygen minimum zones, freshwater and hypersaline lakes, rivers, groundwater, marine surface and deep subsurface sediments, agricultural and forest soils, peats, permafrost, deserts, animal hosts and feces, plant leafs and rhizospheres, salt marshes, bioreactors, processed food, methane seeps, mine drainages, sewages, hydrothermal vents, and hot springs (overview in S1 Data). Particular effort was put into representing soils (14,242 samples across 100 studies), sediments (3,198 samples across 37 studies), and animal guts (8,646 samples across 52 studies), which likely harbor a large fraction of Earth's prokaryotic diversity [22]. Sequences in this composite data set cover at least 200 basepairs in the V4 hypervariable region of the 16S gene, a commonly targeted region in microbial ecology [22–24]. By clustering the pooled sequences at 97% similarity, a commonly used threshold in microbial ecology [2,5,6,9], we recovered hundreds of thousands of OTUs. Based on the recovered OTUs, henceforth referred to as Global Prokaryotic Census (GPC), and through comparisons to previous surveys and existing databases, we estimate global prokaryotic OTU richness and highlight major implications for microbial ecology and evolution. We emphasize that our main objective was to estimate global prokaryotic richness using as deep of a census and covering as many environments and geographic locations as possible; as a trade off, our data set does not offer the same level of experimental standardization across samples nor the amount of metadata included in projects such as the Earth Microbiome Project (EMP) [22].

Here, we focus on OTUs clustered using the conventional 97% similarity threshold so as to facilitate comparison with existing prokaryotic richness estimates [2,5,6,9]. Recent work, however, suggests that a greater similarity threshold (approximately 99%) is often required for distinguishing ecologically differentiated organisms [25–28]. We thus also repeated our analyses using a 99% clustering threshold, which yielded qualitatively comparable results. That said, we point out that clusters of the 16S gene—regardless of similarity threshold and even if completely free of sequencing errors—only provide an approximate "species" analog to sexually reproducing organisms. Indeed, even strains with identical 16S sequences may exhibit different genomic content and ecological strategies; hence, the 16S gene is not always sufficient for distinguishing ecologically differentiated organisms, even when considering exact sequence variants [29–30]. Whether and how prokaryotic "species" can—or even need to—ever be reasonably defined remains highly debated [30–33]. To date, the 16S gene remains an important and the most popular marker for cataloguing prokaryotic diversity and for describing evolutionary relationships in a well-defined and reproducible manner [4,27]. We stress that prokaryotic 16S diversity detected and estimated based on amplicon sequences, as in this and most previous studies, is limited to clades detectable by the PCR primers used. As discussed below, the GPC partly resolves the issue of limited primer scope by using multiple alternative primers; however, it is in principle still possible that some clades are completely missed.

## Results and discussion

### The GPC covers the bulk of global 16S diversity

**Fig 1:**
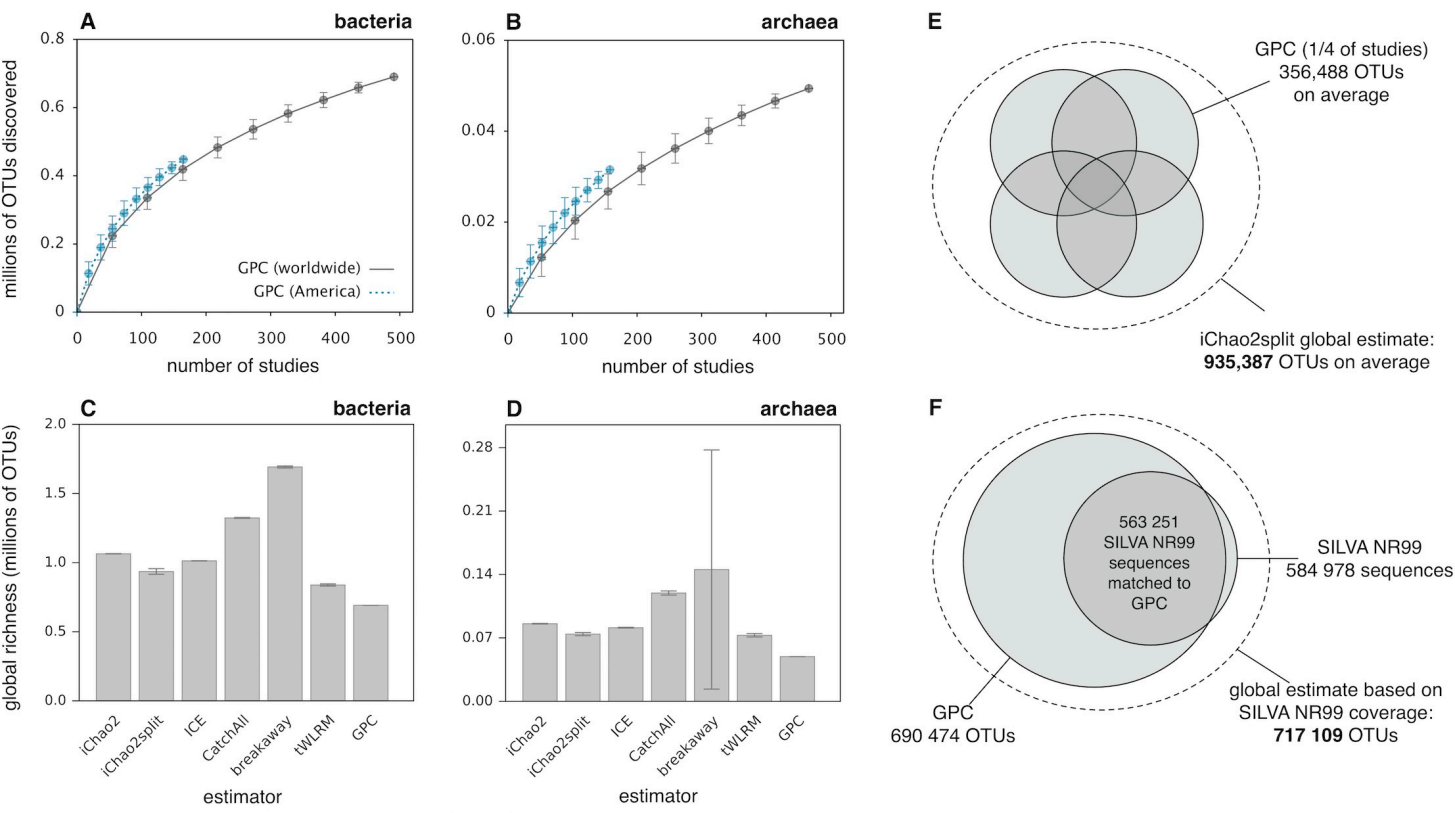
Estimating global prokaryotic OTU richness. (A, B) Accumulation curves showing the number of bacterial (A) and archaeal (B) OTUs discovered, depending on the number of distinct studies included. Curves are averaged over 100 random subsamplings, and whiskers show corresponding standard deviations. Continuous curves were calculated using all studies (worldwide), while blue dashed curves were calculated using solely studies performed in the Americas or near American coasts. (C, D) Global OTU richness of Bacteria (C) and Archaea (D), estimated using the iChao2, iChao2split, ICE, CatchAll, breakaway, and tWLRM estimators. The number of OTUs discovered by the GPC is included for comparison (last bar). Whiskers indicate standard errors, estimated from the underlying models; most standard errors are likely underestimated by the models, so the variability between models is probably a more honest assessment of uncertainty. (E, F) Illustration of two methods used to estimate global bacterial OTU richness (dashed circle). (E) The iChao2split richness estimator is based on the numbers of OTUs discovered once, twice, thrice, or four times when studies are randomly split into four complementary "sampling units" (shaded circles). Average estimates were obtained by repeating the random split multiple times. (F) Based on the fraction of bacterial nonredundant (NR99) sequences in SILVA (right shaded circle) that could be matched to the GPC (left shaded circle), we estimated the fraction of global bacterial OTU richness represented in the GPC and, given the total number of bacterial OTUs in the GPC, the total number of extant bacterial OTUs. For analogous results at 99% clustering similarity, see S4 Fig. GPC, Global Prokaryotic Census; ICE, incidence coverage-based estimator; NR, nonredundant; OTU, operational taxonomic unit; tWLRM, transformed weighted linear regression model.

To ensure maximal phylogenetic coverage, the raw sequencing data from each study was considered as input to our analyses. After stringent quality- and chimera-filtering, the data set comprised 1,734,042,763 high-quality reads, which were pooled and clustered into OTUs at 97% similarity. To avoid spurious (i.e., nonbiological) OTUs generated by sequencing errors or PCR chimeras, only OTUs found in at least two samples of the same study were kept. While this additional quality filter may also remove some biological OTUs, aggressive filtering is necessary for eliminating spurious OTUs, a common and serious problem in amplicon sequencing studies [34–37]. The resulting GPC comprises 739,880 prokaryotic OTUs (690,474 bacterial and 49,406 archaeal), accounting for 1,349,766,275 reads. Accumulation curves of bacterial and archaeal OTUs discovered by the GPC, as a function of studies included, clearly show a deceleration with increasing number of studies (Fig 1A and 1B) and provide an estimate of how many novel OTUs would be discovered in subsequent studies. Specifically, on average, about 93% of bacterial OTUs and 83% of archaeal OTUs found in any additional study are expected to be already included in the GPC. As we show below, this estimate is consistent with the fractions of other independent data sets and databases covered (rediscovered) by the GPC. Most OTUs were matched by at least three reads (88%) and most were found in at least three samples (81%, S2 Fig). Based on the fraction of reads matched to the rarest OTUs (i.e., with only two reads), we estimate that any new random 16S amplicon sequence (i.e., from a randomly chosen prokaryotic cell) would hit an OTU in the GPC at 97% similarity with a probability ≥99.98% (using the Good–Turing frequency formula [38]; see Methods for details). This probability is sometimes referred to as "Good's coverage" and corresponds to the proportion of living or recently deceased prokaryotic cells, detectable by current 16S amplicon sequencing techniques, which is represented by OTUs in the GPC. We emphasize that Good's coverage should not be interpreted as the fraction of global OTU richness represented by the GPC; indeed, estimation of the latter requires additional statistical reasoning, as presented below.

To estimate the total number of extant prokaryotic OTUs globally (discovered plus undiscovered), we used statistical approaches based on the number of OTUs that have been discovered in exactly one study (*Q*_1_), the number of OTUs discovered in exactly two studies (*Q*_2_), and so on. Indeed, the recommended (and only statistically admissible) way to estimate OTU richness is by modeling the incidence frequency counts *Q*_*i*_ in order to predict the number of unobserved OTUs *Q*_0_ [21,39–41]. These methods date back to mathematical theorems for cryptographic analyses during World War II and have been used for microbial as well as macrobial richness estimates [40, 42–44]. Intuitively, widely distributed and abundant OTUs—which are almost certain to be detected—contain very little information about undetected OTUs, while rarely detected OTUs (e.g., detected only once or twice) carry the most information about undetected OTUs; hence, estimators typically rely on the low-frequency counts *Q*_1_, *Q*_2_, etc. [40]. To ensure the robustness of our estimations, we considered several alternative estimation methods, each of which is based on a different frequency model and relies on different assumptions: the improved-Chao2 ("iChao2") richness estimator [45], based on the frequency counts *Q*_1_–*Q*_4_; the incidence coverage-based estimator (ICE) [41], based on the frequency counts *Q*_1_–*Q*_10_; the CatchAll estimator [46], based on frequency counts *Q*_1_–*Q*_τ_, in which τ is chosen adaptively based on internal quality criteria; the transformed weighted linear regression model (tWLRM), which uses a linear regression model for the ratios of consecutive log-transformed frequency counts to predict *Q*_0_ [46,47]; and the breakaway estimator [48], based on a nonlinear regression model for the ratios of consecutive frequency counts. All of the above estimators have been designed to account for heterogeneities in detection frequencies among OTUs (i.e., the presence of rare and frequent OTUs), and breakaway is particularly optimized for efficiently dealing with high fractions of undiscovered diversity. We note that the majority of existing richness estimators, including the ones described above, are based on models in which individual sampling units are assumed to be equivalent (e.g., of the same "effort"); however, studies included in the GPC differ in terms of the environment sampled and the techniques used. To check whether our estimates are sensitive to this caveat, we also deployed an estimation approach whereby we randomly assigned studies to four complementary and equally sized groups (representing four statistically equivalent global "sampling units") and used the iChao2 estimator based on the number of OTUs found in exactly one, two, three of four sampling units ("iChao2split," illustration in Fig 1E). All of the above methods yielded comparable estimates for global prokaryotic OTU richness, with the lowest estimate obtained using tWLRM (901,902 OTUs), and the highest estimate obtained using breakaway (1,588,567 OTUs). The majority of prokaryotic OTUs are estimated to be bacterial, with bacterial richness (Fig 1C) being roughly 10 times greater than archaeal richness (Fig 1D). Importantly, all of the above estimates suggest that there only exist in the order of approximately 1–2 million prokaryotic OTUs, a substantial portion of which is represented by the GPC (47%–82%). We point out that even at a finer phylogenetic resolution (99% clustering similarity), we estimate that there exist only approximately 3–9 million prokaryotic clusters worldwide (S4 Fig and S1 Table), which is six orders of magnitude lower than estimated previously via extrapolation of empirical scaling laws [6].

To further scrutinize our estimates of global OTU richness and to verify whether a substantial fraction of that richness is indeed covered by the GPC, we determined the fraction of 16S sequences from previous global surveys or existing databases that was rediscovered ("recaptured") by the GPC. We found that at 97% similarity, the GPC recaptured 96% of prokaryotic sequences in the SILVA database (nonredundant set, release 132) [14], 89% of prokaryotic sequences in the Ribosomal Database Project (RDP release 11) [12], and 93% of prokaryotic sequences in the Genome Taxonomic Database (GTDB release 86.1) [49] (domain-specific coverages in S2 and S3 Tables). Using these coverages as a proxy for the fraction of global OTU richness covered by the GPC and combining this coverage fraction with the total number of OTUs in the GPC yields additional independent estimates of global prokaryotic OTU richness (771,234–832,420 OTUs, Fig 1F), roughly consistent with our previous estimates. We also found that at 97% similarity, the GPC recaptured 92% of unique noise-filtered ("deblurred") 16S amplicon sequences from another recent independent massive global survey, the EMP [22]. The high fraction of EMP sequences recaptured by the GPC further supports our conclusion that the GPC covers a substantial portion of extant prokaryotic OTUs.

### Eliminating potential caveats

**Fig 2:**
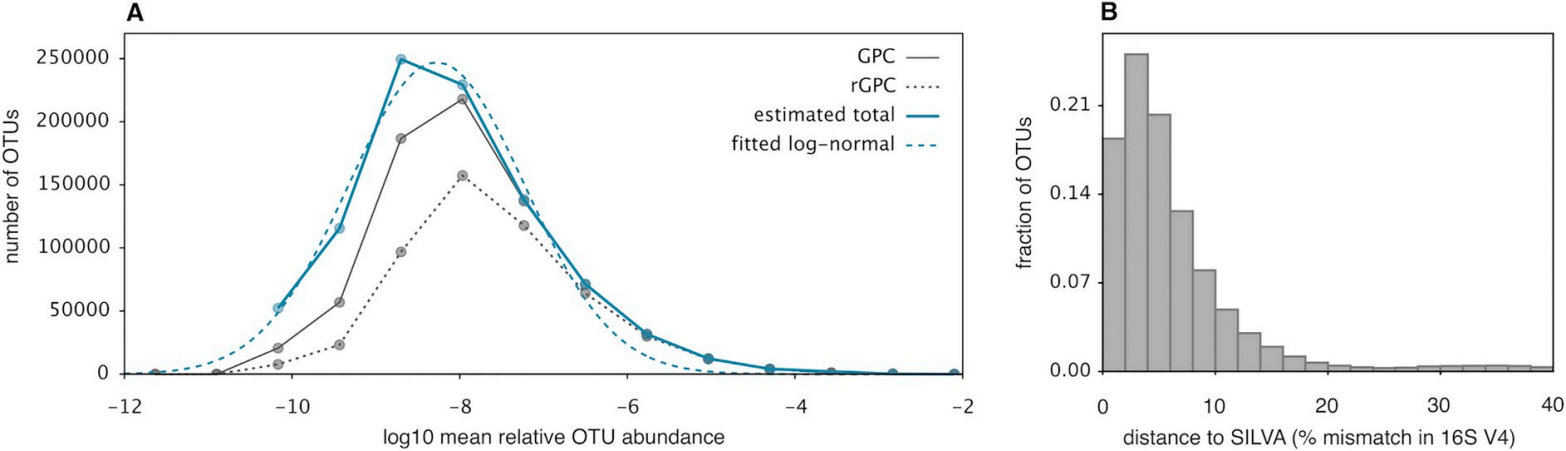
Mean relative OTU abundances and OTU distances to SILVA. (A) Frequency histogram of MRAs of prokaryotic OTUs discovered by the GPC (grey continuous line), of OTUs discovered by the rGPC (grey dashed lines), and of all extant OTUs as estimated using a probabilistic model of OTU discovery (blue continuous curve). The probabilistic model was fitted separately for each MRA interval by comparing the discovery rates of the GPC and the rGPC. The blue dashed curve shows a log-normal distribution model [11] fitted to the estimated MRA distribution of extant OTUs. For analogous results at other phylogenetic resolutions (99%, 95%, and 90% similarity), see S3 Fig. (B) Frequency histogram of the phylogenetic distances of GPC's prokaryotic OTUs (97% similarity in the 16S-V4 region) to SILVA (release 132, set NR99). The distance of an OTU to SILVA was defined as the minimum difference (fraction of nucleotide mismatches over the aligned 16S region) of the OTU to any entry in SILVA. Observe how almost all OTUs have a distance to SILVA below 20%. GPC, Global Prokaryotic Census; MRA, mean relative abundance; NR, nonredundant; OTU, operational taxonomic unit; rGPC, rarefied variant of the GPC.

While our statistical richness estimators (Fig 1C and 1D) were designed to account for variable detection probabilities among OTUs, the potential risk of neglecting a large number of extremely rare OTUs cannot be overemphasized. To further assess this risk, we also explicitly investigated the global distribution of relative OTU abundances. Specifically, for each OTU, we estimated its relative abundance in each sample (using the Good–Turing formula) [38] and then took the average across all samples to obtain its mean relative abundance (MRA). We then created a frequency histogram of MRAs by grouping OTUs into equally sized MRA intervals on a logarithmic axis. We note that this empirical histogram only includes OTUs discovered by the GPC and may thus be skewed toward more abundant OTUs. We therefore reconstructed the total number of extant OTUs in each MRA interval (blue continuous curve in Fig 2A) using a probabilistic model of OTU discovery. This model accounted for our quality filtering and finite sequencing depths and was calibrated by comparing OTU discovery rates in the GPC with those in a rarefied variant of the GPC (i.e., using only half of the original sequences). Following recommendations by Shoemaker and colleagues [11], we then fitted a log-normal model to the reconstructed distribution of MRAs of extant OTUs. We found that the latter was well described by the log-normal model (blue dashed curve in Fig 2A), resembling analogous observations commonly made for larger organisms. We point out that the log-normal model is largely phenomenological, although it is sometimes derived from certain stochastic population models [50]. Hence, we make no assertion as to which mechanisms could possibly lead to the observed log-normal–like distribution of MRAs and as to whether other (potentially yet to be discovered) models may be even more suitable. Based on the reconstructed distribution of MRAs, as well as based on the fitted log-normal model, we estimate that the majority of extant OTUs exhibit an MRA across samples between 5 ×10^-10^ and 5 ×10^-8^ (mode approximately 5 ×10^-9^). For lower MRAs, the number of OTUs declines rapidly toward zero. The rapid decline of the number of OTUs for lower MRAs suggests that the number of much more rare OTUs (specifically, with an MRA lower than the OTUs detected by the GPC) is relatively small and that the GPC did not miss vast numbers of extremely rare OTUs. This conclusion contrasts previous speculations that there exists a vast number of extremely rare and largely undetected OTUs, sometimes referred to as "rare microbial biosphere" [6,17,51]. According to the fitted log-normal model, there exist only approximately 886,291 prokaryotic OTUs across the entire range of MRAs, further supporting our other estimates.

Since OTUs are inevitably taxonomically identified through comparison with reference databases (here, SILVA was used to identify OTUs at the kingdom level), censuses such as the GPC may in principle miss clades lacking a close relative in the databases. To investigate this potential caveat, we calculated the phylogenetic distance of each OTU to its closest match in SILVA in terms of 16S sequence divergence and created a frequency histogram of these distances that shows the overall distribution of OTUs in comparison to SILVA (Fig 2B). We found that the vast majority of OTUs in the GPC has a distance to SILVA that is far below the threshold allowed for taxonomic identification (maximum 40%) and that the frequency of OTUs drops rapidly toward that threshold. This suggests that our taxonomic identification algorithm did not miss a substantial number of biological sequences at larger phylogenetic distances (omitted sequences at greater distances are likely spurious, see Methods for details).

Primer "blind spots," i.e., clades not captured by PCR primers, could in principle lead to an underestimation or a phylogenetically biased assessment of prokaryotic diversity by the GPC. For example, recent studies suggest that roughly 10% of prokaryotic 16S sequences may be missed by any given existing primer pair [52–54]. To investigate this caveat and to check whether a large fraction of diversity may have been missed by the GPC due to primer blind spots, we calculated the fraction of 16S sequences recovered from a multitude of environments using primer-independent (metagenomics-based) methods that were rediscovered by the GPC. We found that, at 97% similarity, the GPC recaptured 91% of 16S sequences in prokaryotic genomes previously assembled from metagenomes (Uncultivated Bacteria or Archaea [UBA]) [55] and 93% of bacterial 16S sequences extracted from thousands of public metagenomes [56]. These recapture fractions are comparable to the fraction recovered from the EMP, suggesting that the fraction of OTUs missed by the GPC due to primer blind spots is small. One reason may be that the GPC comprises sequences obtained using a multitude of alternative primers optimized for different clades, therefore partly alleviating the problem of primer nonuniversality. In particular, 16S sequences currently not detectable by any primers may only represent a minority of prokaryotic diversity, even if any given primer set has limited sensitivity scope. It is thus improbable that primer-independent methods will reveal a prokaryotic richness much (i.e., orders of magnitude) higher than composite multiprimer-based surveys such as the GPC.

### Most prokaryotic OTUs are globally distributed

When we repeated our analyses using only studies from the Americas or near American coasts (165 studies across 14 countries, see map in S1 Fig) instead of the full GPC, OTU discovery rates for any given number of studies remained almost unchanged (Fig 1A and 1B). Hence, for the same "sampling effort," the same OTU richness is recovered from the Americas as from the full GPC, and importantly, the restriction to the Americas does not cause a stronger deceleration of OTU discovery rates. This suggests that the majority of global prokaryotic OTUs could have been censused from a single hemisphere, if sufficient samples had been available. Consistent with this conclusion, when controlling for the number of studies included and using the same methods as above, we found that prokaryotic OTU richness estimated for the Americas was very similar to estimates based on an equal number of studies randomly chosen from across the world (0.7–1.3 million OTUs, S5A Fig). Similar results were also obtained at a higher 16S similarity threshold of 99% (S4A and S4B and S5B Figs). Our findings extend previous observations that for any given number of samples, similar prokaryotic OTU richness is recovered from soil in New York Central Park as from distinct soil samples worldwide [57]. Most prokaryotic OTUs thus appear to exhibit low geographic endemism and global dispersal ranges at geological time scales, i.e., at time scales needed for 16S to diverge by more than 1% [58,59]. A global distribution of prokaryotic OTUs has long been a central but controversial hypothesis [60,61]. Our finding provides strong support for this hypothesis and is also consistent with previous findings that most marine bacterial OTUs can be recovered from a single location in the ocean with sufficiently deep sequencing [62,63] and with findings that salt-marsh Nitrosomonadales OTUs are globally distributed [64]. That said, we point out that a global distribution of OTUs does not rule out geographic endemism at finer phylogenetic resolutions since younger clades, e.g., recently differentiated ecotypes with identical 16S, may not have had time to overcome dispersal barriers at global scales [65,66].

### Taxon-specific diversities and coverages in databases

**Fig 3:**
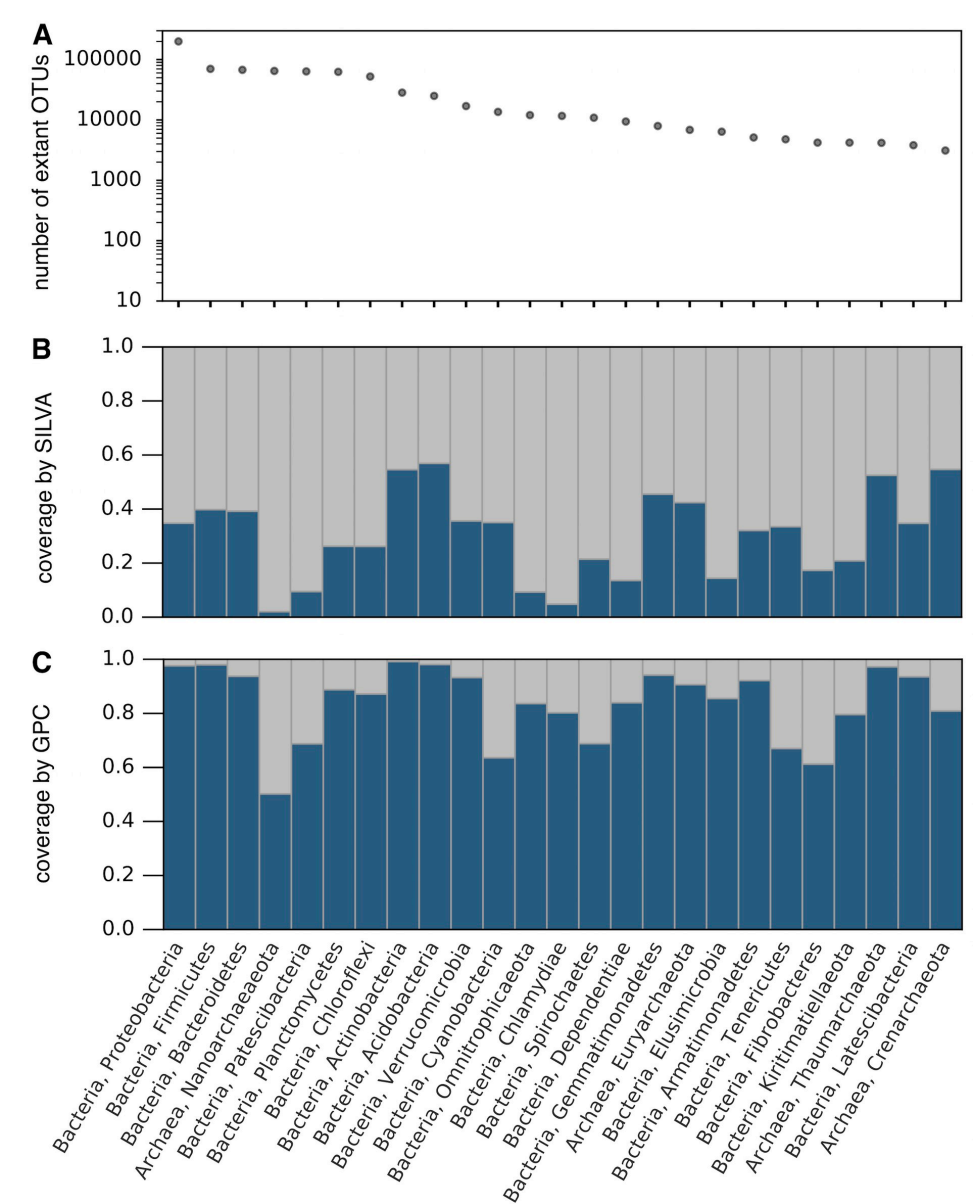
Richness of phyla and coverage by SILVA and the GPC. (A) Estimated number of OTUs (97% similarity in the 16S-V4 region) globally, within various prokaryotic phyla. Estimated based on the coverage of SILVA by the GPC (subfigure C) and the number of OTUs in the GPC. Only phyla including at least 10 entries in SILVA (release 132, set NR99) [14] and estimated to contain at least 10 extant OTUs are considered. Phyla are sorted in decreasing estimated OTU richness; only the 25 richest phyla are shown. (B) Fraction of GPC OTUs that could be mapped to SILVA NR99 at similarity ≥97%, as a proxy for global OTU richness covered by SILVA, within the same phyla as in A. (C) Fraction of SILVA NR99 sequences that could be mapped to the GPC at similarity ≥97%, as a proxy for global OTU richness covered by the GPC, within the same phyla as in A. For additional phyla not shown here, see S6 Fig. For analogous results at other phylogenetic resolutions, see S7 Fig (99%), S8 Fig (95%), and S9 Fig (90%). For analogous results at the class level, see S10 Fig (99%), S11 Fig (97%), and S12 Fig (95%). GPC, Global Prokaryotic Census; NR, nonredundant; OTU, operational taxonomic unit.

Our census allows an unprecedentedly precise assessment of the diversity covered by existing 16S databases such as SILVA [14] or the RDP [12]. Based on the fraction of GPC OTUs matched to entries in SILVA (release 132, nonredundant set) at 97% similarity, we estimate that SILVA represents about 29% of bacterial and 14% of archaeal OTUs globally. Similarly, we find that the RDP (release 11) represents about 42% of bacterial and 20% of archaeal OTUs. Our findings confirm recent estimates that SILVA covers about 30%–40% of global prokaryotic OTU richness [5,67]. We point out that Bacteria are currently overrepresented in SILVA and the RDP relative to Archaea. The uneven representation of various taxonomic groups is generally more pronounced at lower taxonomic levels, with some phyla being strongly overrepresented compared to others (Fig 3B). In addition, about 7% of prokaryotic OTUs in the GPC could not be reliably assigned to any phylum listed in SILVA. This indicates that some phyla are not represented in SILVA at all, consistent with conclusions from metagenomic studies [56,68].

Our estimates also highlight strong differences in the OTU richness specific to different phyla, with Proteobacteria (mostly Gammaproteobacteria and Deltaproteobacteria) clearly dominating global richness, followed by the Firmicutes (mostly Clostridia), Bacteroidetes (mostly Bacteroidia), Nanoarchaeota (mostly Woesearchaeia), Patescibacteria, and Planctomycetes (mostly Planctomycetacia) (Figs 3A and S11A). Hence, the large representation of Proteobacteria in reference databases and among cultured species [69] is not just the result of a biased discovery rate (e.g., due to ease of culturing) but also partly reflects their general ability to expand and persist in a multitude of ecological niches [70]. Similarly, the large richness of Firmicutes may be explained by their ability to colonize a wide range of animal hosts [56]. Interestingly, the Nanoarchaeota are known as a deeply branching and poorly characterized ancient clade [71], which has been suggested to comprise a largely underestimated diversity [72]. The few isolated Nanoarchaeota indicate that they share a common history of adaptation to ectosymbiosis [73], and this may have contributed to the difficulty of isolating representatives. In contrast, while the Actinobacteria phylum contains the second largest number of cultured strains [69,74], it only ranks eigth in terms of estimated total OTU richness (Fig 3A), suggesting a strong culturing bias for this phylum, consistent with previous findings [69]. We point out that extant prokaryotic diversity is the result of diversification and extinction processes operating over billions of years and throughout geological transitions [15]. It is thus possible that the relative richness of various taxa varied strongly over time.

### Implications

Our work suggests that global prokaryotic OTU richness is about six orders of magnitude lower than previously predicted via extrapolation of diversity scaling laws and OTU abundance distributions fitted to individual microbial communities [6,8]. While we find support for a log-normal distribution of mean relative OTU abundances consistent with assumptions made by Locey and colleagues [6], at least two aspects differentiate our approach from Locey and colleagues. First, we fitted the log-normal model to a global data set comprising thousands of samples across hundreds of environments rather than to individual local communities, thus obtaining a description of relative abundances that is more suitable for global richness estimates. Second, we did not assume or extrapolate any phenomenological scaling relationships between different parameters of the model, thus relying on fewer questionable assumptions. The discrepancy between our estimates and those by Locey and colleagues [6] suggests that phenomenological scaling relationships of microbial diversity cannot be extrapolated to global scales when these relationships were fitted solely to individual communities. This conclusion also supports arguments by [21] that the extrapolations performed by Locey and colleagues [6] have no predictive power and are statistically unsound. Our estimates also contrast extrapolations by Larsen and colleagues [9], who argued that there exist billions of animal-associated bacterial OTUs based on the number of OTUs typically found in individual insect species and the estimated total number of animal species. One reason for this discrepancy may be that Larsen's extrapolation did not properly account for the overlap of microbiomes between animal taxa (detailed discussion in S1 Text). Our much lower bacterial richness estimates suggest that many symbiotic OTUs are found in multiple host species that may or may not be closely related, potentially due to host trait convergences, consistent with recent observations [75–77]. Since the microbiome of only a minuscule fraction of animal species has been examined so far, it is quite possible that many allegedly "host-specific" bacteria are shared by a broader spectrum of host species than currently known. This could explain why overall bacterial richness (at the OTU level) appears to have been largely unaffected by past mass animal extinctions, as recently suggested based on phylogenetic analyses [15].

Given the long evolutionary history and ubiquity of prokaryotes, a richness of only approximately 0.8–1.6 million OTUs may seem surprisingly low. To put this finding into perspective, we considered a steady state null model, in which global prokaryotic cell counts (*N*) are constant over time, in which cells are replaced randomly and regardless of phylogenetic relationships via births and deaths, and in which the 16S-V4 region evolves neutrally [59] at some constant drift rate (*r*, measured in mutations per site per generation) and independently at each site. Note that one important and potentially wrong assumption of this model is that cell turnover is statistically independent of phylogeny. A similar model was recently proposed by Straub and colleagues [78] as a null model for 16S phylogenies. Based on our model, we predict that there should exist about $2rN/0.03∼10^{22}-10^{23}$ OTUs (assuming *N* = 10^30^ [79] and $r=4\times 10^{-9}-5\times 10^{-10}$ [80,81], details in S2 Text). This extreme discrepancy between the model and our global richness estimates persists regardless of the similarity threshold used (97% or 99%). The discrepancy also persists even if currently estimated 16S mutation rates (*r*) or global cell counts (*N*) were off by 10 orders of magnitude or even if global cell counts varied drastically (e.g., by 1–10 orders of magnitude) over recent time. One explanation for this discrepancy could be that the evolution of the 16S-V4 region along a lineage is subject to strong constraints that favor some mutations or sequence variants more than others, thus effectively reducing the "permissible" sequence space [82–84]. This would suggest that only about 10^−14^% of the theoretically possible 16S variants are actually biologically viable and attainable over the course of approximately 4 billion years [85]. Alternatively, some processes not captured by the model may eliminate all but just a small fraction of 16S sequence variants emerging over time. Phylogenetically correlated turnover, i.e., closely related organisms experiencing birth or death simultaneously more frequently than expected by chance (e.g., due to their greater ecological similarity), would lead to increased removal of sequence variants from the pool compared to the above null model and may also be an explanation for the relatively sparse filling of 16S sequence space found here. This would imply that extinction plays a central role in prokaryotic diversification, as recently suggested by [15] and contrasting common speculations that prokaryotic OTUs are unlikely to go extinct [1,86–88].

We emphasize that our results are specific to the similarity threshold used (97% similarity in 16S) and the gene region targeted (V4), although these choices are a popular combination in microbial ecology [22]. For example, at coarser phylogenetic resolutions (e.g., 95% and 90% similarity, roughly corresponding to genera and families [89,90]), we estimate that there exist substantially fewer 16S clusters and that the GPC covers a greater fraction of those clusters (50%–98% and 51%–99.5% of extant clusters, respectively, S1 Table). Consistent with these estimates, we found that at these coarser resolutions the GPC recaptured 95%–96% and 98%, respectively, of previous global 16S surveys (S3B, S3C, S8 and S9 Figs and S4 and S5 Tables). Reciprocally, when we analyzed a subset of our data (approximately 0.2 billion reads across 111 studies) at the finest possible phylogenetic resolution (100% identity) using the recent Divisive Amplicon Denoising Algorithm (DADA2) software [91], we obtained about 3.4 times as many exact amplicon sequence variants (ASVs) as 97%-OTUs and about 1.5 times as many ASVs as 99%-OTUs (S13 Fig). This suggests that the global richness of exact sequence variants is at most an order of magnitude larger than the number of OTUs. The sequence length considered may also affect global richness measures. For example, full-length 16S diversity (currently much harder to census) is expected to be greater than partial-length (V4) 16S diversity [4] because short gene regions may cluster as one OTU due to the stochasticity of mutations even if the full genes differ by more than 3%. For example, when restricted to the V4 region or when considering the full 16S gene, at 97% similarity, 16S sequences in SILVA cluster into 102,416 or 270,788 OTUs, respectively, suggesting that the number of extant full-length OTUs may exceed the number of V4 OTUs by a factor of approximately 2.7. When combined with our V4-based richness estimates, this suggests that there exist 2.2–4.3 million full-length OTUs worldwide. A similar ratio between full-length and partial-length clusters is also obtained at 99% similarity (S6 Table). Unfortunately, while full-length sequencing undoubtedly improves phylogenetic resolution, technical complications and a higher cost currently prevent the wide adoption of full-length 16S sequencing in microbial community surveys. Finally, we stress that 16S diversity only provides a coarse surrogate for prokaryotic genomic and phenotypic diversity [29,30], and it is probable that the global number of prokaryote ecotypes greatly exceeds the number of OTUs. Cataloguing the phenotypic and genomic diversity of prokaryotes will undoubtedly be an important but much more challenging future task.

## Conclusions

In 2002, Curtis and colleagues [2] hypothesized that experimental approaches to directly enumerating extant prokaryotic diversity will remain fruitless due to logistical challenges. Almost two decades later, we demonstrated that publicly available sequencing data from 492 studies around the world are sufficient to recover a substantial fraction (47%–96%) of global prokaryotic diversity in the 16S-V4 region, the very extent of which has long been a topic of speculation [1,2,4–7]. Our composite data set, covering a multitude of environments worldwide, enabled us to strongly constrain global prokaryotic OTU richness. Indeed, our global richness estimates are similar across a multitude of statistical estimators (Fig 1C and 1D), all of which are based on different models of OTU detection probabilities and, in most cases, use a different set of OTU incidence frequency counts. The high fraction of 16S sequences from other amplicon- and metagenomic-sequencing surveys (e.g., the EMP [22] or UBA [55]) and large databases (e.g., SILVA [14] and RDP [12]), recaptured independently by the GPC (91%–93%), further supports our global prokaryotic richness estimates and our assessment that the GPC covers a substantial portion of that richness.

While no particular 16S similarity threshold provides an ideal species analog, OTUs provide an operational and clearly defined measure of richness that can be compared across studies, environments, and geological time [15]. For example, our work revealed that global prokaryotic OTU richness is orders of magnitude lower than often predicted [1,6,9], regardless of the considered similarity threshold (97% and 99%). Further, the fact that our global richness estimates are approximately 16–17 orders of magnitude lower than predicted by a null model for neutral OTU emergence, regardless of the similarity threshold used, suggests that extinction played a major role in prokaryotic evolution [15] and/or that the attainable 16S-V4 sequence space is extremely constrained. Our work also showed that at the phylogenetic resolutions considered here (≥1% divergence in 16S), most prokaryotic OTUs are globally distributed, yielding insight into the time scales involved in global-scale microbial dispersal.

We reiterate that the goal of the GPC was to enable a more robust estimate of total extant prokaryotic richness than previous studies. Indeed, our estimates are based on an unprecedentedly large and environmentally broad composite sequencing data set, assembled from hundreds of studies utilizing alternative primers and alternative sampling techniques, and using a wide array of alternative statistical estimation methods for increased robustness. The GPC can thus facilitate future efforts to catalogue and phenotypically describe Earth's extant prokaryotes. The GPC also opens up new avenues for reconstructing prokaryotic evolution over geological time using massive phylogenetic trees and for refining macroecological theories. While long considered an unseen majority [79], thanks to ongoing technological revolutions, prokaryotes could one day become one of the most exhaustively characterized and best understood forms of life.

## Methods

### Retrieval of GPC amplicon sequences

Publicly available 16S rRNA amplicon sequences (V4 region) from various environmental and clinical studies were downloaded from the European Nucleotide Archive (https://www.ebi.ac.uk/ena). Only Illumina sequences were downloaded to ensure sequence qualities en par with current standards and because Illumina-based studies typically achieve much deeper sequencing than studies using previous-generation (e.g., 454) technology. We only considered sequences covering the V4 hypervariable region for three reasons. First, use of the same gene region in all samples is necessary for clustering sequences into nonredundant OTUs. Second, the V4 region is one of the most popular regions targeted in microbial surveys, including the EMP [22], making it easier to find publicly available data sets and allowing for comparison with the EMP. Third, the V4 region was shown to be the most suitable single hypervariable region for reconstructing bacterial phylogenetic relationships [24]. Studies were chosen to represent as wide of an environmental spectrum as possible. A total of 34,368 samples from 492 studies were downloaded (description and accession numbers in S1 Data). Geographical sample locations (where available) are shown in S1 Fig.

We mention that sequencing data from the EMP [22] were omitted from the GPC because this allowed us to use the EMP as an independent reference data set for assessing the fraction of OTUs rediscovered by the GPC and because the much shorter read lengths in the EMP (122 bp on average) compared to the GPC (246 bp on average) would reduce the available phylogenetic resolution [92–96]. Indeed, as we expected the EMP to be less phylogenetically biased than reference databases such as SILVA and RDP, the EMP provided a valuable means to further evaluate the overall coverage of extant prokaryotic diversity by the GPC (see main text and Methods below).

### Amplicon sequence clustering

Paired-end reads with sufficient overlap were merged using flash v1.2.11 [97] (options—min-overlap = 10—max-mismatch-density 0.01—phred-offset 33—allow-outies). Of the nonsufficiently overlapping pairs, forward reads were kept and reverse reads discarded. Single-end reads, merged paired-end reads, and nonmerged forward reads were subsequently processed in the same way, as follows. Reads were trimmed and quality filtered using vsearch v2.6.2 [98], keeping only reads that were at least 200 bp long after trimming (options—fastq_ascii 33—fastq_minlen 200—fastq_qmin 0—fastq_maxee 0.5—fastq_truncee 0.5—fastq_maxee_rate 0.002—fastq_stripleft 7—fastq_trunclen_keep 250—fastq_qmax 64). Any samples with more than 10^6^ quality-filtered reads were subsampled down to 10^6^ randomly chosen reads to reduce computational requirements; samples with fewer quality-filtered reads were not subsampled. The 1,988,445,238 kept reads were then chimera-filtered de novo using vsearch (options—abskew 1.9 –mindiv 0.5 –minh 0.1) separately for each sample. About 10% of reads were identified as chimeric (on average, 8.6% of reads per sample), yielding in total 1,734,042,763 quality-filtered and chimera-filtered reads with a mean length of 246 bp. Reads from all samples were pooled and subsequently clustered de novo at 97% similarity using cd-hit-otu v0.0.1 [99]. We chose cd-hit-otu because—in contrast to most other OTU-clustering algorithms—it scales relatively well to massive data sets such as ours. For a comparison between cd-hit-otu and other clustering algorithms, we refer to [99–102]. For consistency with our own downstream error filters (removal of spurious OTUs), we set the minimum size for a cluster of duplicates in the cd-hit-otu algorithm to 2 (step clstr_sort_trim_rep) and the primary cluster size cutoff to 1 (disabling cd-hit-otu's noise removal algorithm). De novo clustering yielded 1,545,602 clusters. Because primers of the various studies included did not all cover exactly the same regions and due to the clustering algorithm implemented by cd-hit-otu, a small number of clusters was redundant, i.e., the representative sequences of some clusters were slightly shifted versions of others. To remove this redundancy, we further clustered representative sequences using vsearch (command—cluster_fast—usersort–id 1.0—iddef 2—strand plus), thereby obtaining 1,386,686 nonredundant OTUs. To further avoid spurious (i.e., nonbiological) OTUs, we only kept OTUs that were found in at least two samples of the same study (944,863 OTUs). While we cannot completely rule out the inclusion of some spurious OTUs in the GPC, we point out that a hypothetical removal of these OTUs would only further decrease our estimates of global prokaryotic OTU richness. Representative sequences for the final set of prokaryotic GPC OTUs (at 97% and 99% clustering threshold) and OTU tables are available online at www.loucalab.com/archive/GPC.

### Taxonomic identification of OTUs

The taxonomic identity of each OTU was determined based on its similarity to entries in the SILVA database [14] and by using a consensus approach, as follows. Each OTU was mapped to SILVA's nonredundant (NR99) SSU sequences using vsearch [98], at a similarity threshold of 60% and keeping only the top 10 hits (options "—id 0.6—strand both—iddef 2—maxaccepts 20—maxhits 10"). If at least one hit had a similarity 100%, then all hits with similarity 100% were used to form a consensus taxonomy. Otherwise, if the best hit had a similarity s≥60%, then all hits with similarity ≥(*s*-5%) were used to form a consensus taxonomy. In either case, the consensus taxonomy of a set of hits was defined as the taxon at the lowest taxonomic possible level (e.g., domain, phylum, etc.) containing all of the hits. If an OTU did not have any hit in SILVA at or above a threshold of 60% similarity or did not have a consensus taxonomy even at the domain level, it was considered unidentified and was subsequently omitted (see justification in the next paragraph). The overwhelming majority (87%) of OTUs had at least one hit in SILVA at similarity ≥60%, and almost all of these OTUs (>99.9%) could be identified at some taxonomic level. Any OTUs identified as eukaryotes, chloroplasts, and mitochondria were omitted from subsequent analyses.

We note that our imposed similarity threshold of 60% to SILVA is much lower than the thresholds commonly suggested for delineating phyla (e.g., 75% similarity according to [4]), thus the bulk of biological (i.e., nonspurious) sequences is expected to pass this threshold. While the 75% similarity threshold by [4] referred to the full-length 16S gene, the same study also showed that partial gene regions (e.g., "R3" in that paper, roughly corresponding to V4) exhibit less richness than the full-length gene for any given clustering threshold. Hence, organisms that are >75% similar to a SILVA entry in the full 16S are even more likely to be >75% similar in the V4 region; consequently, a similarity threshold of 60% in V4 is probably more permissive than a similarity threshold of 75% in the full gene. OTUs with a similarity to SILVA below 60% (or equivalently, a distance above 40%) are likely largely spurious. To confirm this expectation and to further investigate the nature of these omitted OTUs, we calculated the distribution of distances of OTUs to SILVA as well as the fraction of OTUs that could be matched to SILVA, as the similarity threshold decreased below 60% all the way to zero (S14A and S14B Fig). We found that as one approaches the 60% similarity threshold, the fraction of OTUs matched to SILVA levels off; that is, very few OTUs lie in the 60%–65% range, while the majority of OTUs lies in the 80%–100% range (as discussed in the main article). Strikingly, for slightly lower similarity thresholds, there exists a sharp peak of OTUs within the 50%–60% similarity range and virtually no OTUs below that range. This agglomeration of a small fraction of OTUs in the 50%–60% range is likely mostly spurious, specifically consisting of bichimeras (the most common type of chimeras). Indeed, bichimeras inevitably include a biological segment that makes up at least 50% of their length, and that biological segment will likely match SILVA at considerable similarity. Thus, most bichimeras are expected to aggregate within the 50%–60% similarity interval, as observed in our case. When we repeated the above analysis for clusters at 99% identity, we observed that the peak within the 50%–60% similarity range decreased substantially (S14C and S14D Fig). This is consistent with the expectation that chimeric sequences clustered at 99% identity are easier to detect than when clustered at 97% identity, since the variance around representative sequences hinders a reliable identification of parent sequences by chimera detectors. In fact, when we considered exact ASVs generated and chimera-filtered with DADA2 [91] for a subset of our data (subset "AG," see below), the peak in the 50%–60% similarity interval disappeared nearly completely (S15C Fig). In other words, almost all ASVs had a similarity to SILVA above 60%. This is consistent with the expectation that chimeric ASVs are easier to detect than chimeric OTUs [91] and further supports our conclusion that most of the removed OTUs (all falling within the 50%–60% similarity interval) are likely bichimeras that have escaped our previous chimera filters. The alternative explanation that this agglomerate at 50%–60% similarity represents biological sequences is much less probable, since this would beg the question as to why these sequences aggregate within the similarity interval 50%–60% and why they disappear at higher clustering identities.

### Comparison with the EMP

To calculate the fraction of prokaryotic 16S diversity recovered by the EMP [22] that was recaptured by the GPC, we proceeded as follows. We dowloaded the EMP's set of unique quality- and chimera-filtered 16S sequences (202,540 "deblurred" sequences, covering 150 bp of the V4 region) from the EMP FTP repository (ftp://ftp.microbio.me/emp/release1/otu_info/deblur/emp.150.min25.deblur.seq.fa). EMP sequences were taxonomically identified using the same methods as for the GPC, and any sequences identified as eukaryotes, chloroplasts, or mitochondria were omitted. EMP sequences were then mapped to GPC OTUs using vsearch at a similarity threshold of 97% whenever possible (options "—id 0.97—iddef 2—strand both"). For any given taxon, the fraction of recaptured EMP sequences was calculated as *N*_m_/*N*_E*MP*_, in which *N*_E*MP*_ is the number of EMP sequences identified to be within the focal taxon and *N*_*m*_ is the number of EMP sequences in the focal taxon matched to a GPC OTU. An overview of recapture fractions is provided in S2 Table.

### Comparison with the RDP

To calculate the fraction of prokaryotic 16S diversity in the RDP (release 11) [12] that was recaptured by the GPC, we proceeded as follows. Nonaligned bacterial and archaeal 16S sequences were downloaded as fasta files from the RDP website (https://rdp.cme.msu.edu/misc/resources.jsp). The RDP's original taxonomic annotations were assumed for each RDP sequence. The fraction of RDP sequences recaptured by the GPC was calculated for various taxa, as described above for the EMP (overview in S2 Table).

### Comparison with the GTDB

To calculate the fraction of prokaryotic 16S diversity in the GTDB (release 86.1) [49] that was recaptured by the GPC, we proceeded as follows. Bacterial and archaeal 16S sequences, extracted from the GTDB genomes, were downloaded as fasta files from the GTDB website (http://gtdb.ecogenomic.org/downloads). Only sequences at least 1,000 bp long were kept. The fraction of GTDB sequences recaptured by the GPC was calculated for various taxa, as described above for the EMP (overview in S2 Table).

### Comparison with UBA

To calculate the fraction of 16S sequences from metagenome-assembled UBA genomes [55] that was recaptured by our GPC data set, we proceeded as follows. Fully or partly assembled 16S sequences for 2,853 metagenome-assembled genomes were downloaded from https://data.ace.uq.edu.au/public/misc_downloads/uba_genomes/ on October 25, 2017. Only UBA sequences longer than 1,000 bp were considered to increase the probability of adequate overlap with the V4 region, leaving us with 620 sequences. UBA sequences were taxonomically identified using the same methods as for the GPC, and any sequences identified as eukaryotes, chloroplasts, or mitochondria were omitted. The fraction of UBA sequences recaptured by the GPC was calculated for various taxa, as described above for the EMP (overview in S2 Table).

### Comparison with IMG/M

To calculate the fraction of bacterial 16S sequences previously extracted from metagenomes in the Integrated Microbial Genomes and Microbiomes (IMG/M) database [56] that was recaptured by the GPC, we proceeded as follows. Aligned SSU sequences (≥1,200 bp long) extracted from IMG/M were downloaded as a fasta file from https://bitbucket.org/berkeleylab/bacterialdiversity/downloads on February 13, 2018 (file IMGG_SSU1200.fasta). Only sequences obtained from metagenomes were kept (tag "MTGBAC," 63,367 sequences). Aligned sequences were dealigned (gaps removed); taxonomically identified, as described above for the GPC; and any sequences identified as eukaryotes, chloroplasts, or mitochondria were omitted. The fraction of IMG/M sequences recaptured by the GPC was calculated for various taxa, as described above for the EMP (overview in S2 Table).

### Comparison with SILVA

Unless otherwise mentioned, sequences in SILVA classified as eukaryotes, mitochondria, or chloroplasts were omitted from all analyses. To calculate the fraction of 16S diversity in the SILVA database [14] that was covered ("recaptured") by the GPC (Figs 3C and S11C), we proceeded as follows. Nonredundant (NR99) SSU alignments in SILVA release 132 were downloaded from the SILVA website (https://www.arb-silva.de/fileadmin/silva_databases/release_132/Exports/SILVA_132_SSURef_Nr99_tax_silva_full_align_trunc.fasta.gz) and subsequently dealigned (gap characters removed). Dealigned SILVA NR99 sequences were then mapped to GPC OTUs via global alignment using vsearch, at a similarity threshold of 97% (options "—id 0.97—iddef 2—strand both"). For any given taxon (domain, phylum, or class), we calculated the coverage by the GPC (Figs 3C and S11C) as the ratio (*ρ*) of mapped SILVA sequences in that taxon divided by the total number of SILVA sequences in that taxon. The total number of extant OTUs within the taxon (Figs 3A and S11A) was estimated as *N*_G*PC*_*/ρ*, in which *N*_G*PC*_ is the number of GPC OTUs in the taxon.

To estimate the coverage of various prokaryotic taxa (domains, phyla, or classes) by SILVA (Figs 3B and S11B), we proceeded as follows. For any given taxon, we mapped GPC OTUs within that taxon to the dealigned SILVA NR99 sequences via global alignment using vsearch at a similarity threshold of 97% (options "—id 0.97—iddef 2—strand both"). The fraction of OTU richness covered by SILVA was estimated as the ratio of mapped GPC OTUs within that taxon divided by the total number of GPC OTUs in that taxon.

To calculate the 16S diversity in SILVA, in terms of OTUs comparable to the GPC (clusters at 97% identity in the V4 region), we proceeded as follows. We downloaded the full set of SSU alignments from the SILVA website (https://www.arb-silva.de/fileadmin/silva_databases/release_132/Exports/SILVA_132_SSURef_tax_silva_full_align_trunc.fasta.gz). We then aligned GPC OTUs to SILVA using the QIIME script parallel_align_seqs_pynast.py [103] and using a random subset (1%) of the SILVA alignments as a template. We identified the first nucleotide position in the GPC alignments that had a gap fraction below 0.9 (*Escherichia coli* position 516) and extracted the part starting at that nucleotide position and extending 200 bp in the 5'→3' direction (excluding gaps) from the SILVA alignments. Extracted partial SILVA alignments were then dealigned (gaps removed) and clustered at 97% similarity using uclust v1.2.22 [104], yielding 102,416 prokaryotic OTUs ("SILVA V4-OTUs"). To calculate the 16S diversity in SILVA in terms of full-length OTUs, we also clustered the full-length dealigned SILVA sequences using uclust at 97% similarity, obtaining 270,788 prokaryotic OTUs.

To calculate the distances between GPC's OTUs and SILVA (Fig 2B), we proceeded as follows. OTUs were globally aligned against SILVA NR99 sequences using vsearch, keeping only the top hit (options "—id 0.6—iddef 2—strand both—maxaccepts 1000—maxhits 1—top_hits_only"). For any OTU, its distance to SILVA was defined as 100−*I*, in which *I* is the percentage identity to the top hit. The histogram in Fig 2B was obtained after binning distances into intervals of 2%.

### Comparing OTUs to ASVs

In order to obtain a rough estimate of the global richness expected in terms of ASVs (which, in the absence of errors, are equivalent to sequence clusters at 100% similarity) when compared to OTU richness—the standard richness measure considered in previous studies—we investigated the density of exact ASVs in the GPC. ASVs were determined using DADA2 v1.10.0 [91], a tool that fits a stochastic error model to the available sequencing data in order to then distinguish between likely sequencing errors and true biological sequence variants. To limit computational requirements, we only considered a pseudo-randomly chosen subset of GPC studies (111 studies with paired-end reads and whose names started with the letter "A" through "G"), henceforth referred to as "AG" subset. (This subset was chosen for convenience of file handling, and an alphabetical choice of projects is practically random for our purposes.) Any samples with more than 10^6^ raw reads were subsampled down to 10^6^ randomly chosen reads to reduce computational requirements. Reads were quality-filtered and trimmed using the DADA2 function filterAndTrim, with options maxEE = 0.5, minLen = 160, truncQ = 0, trimLeft = 7, truncLen = 167 for forward reads and options maxEE = 1, minLen = 140, truncQ = 0, trimLeft = 7, truncLen = 147 for reverse reads. This yielded 357,738,981 quality-filtered nonmerged paired-end reads. Error rate models were fitted using the DADA2 function learnErrors, separately for each study and separately for forward and reverse reads. ASVs were then inferred for each sample using the DADA2 functions derepFastq and dada (with options pool = FALSE, selfConsist = FALSE), and paired-end denoised reads were subsequently merged using the DADA2 function mergePairs (with options minOverlap = 10, maxMismatch = 0). A preliminary ASV table was created using the DADA2 function makeSequenceTable, yielding an ASV table comprising 258,448,458 reads across 2,319,542 ASVs. Chimeric sequences (specifically, bichimeras) were subsequently removed using the DADA2 function removeBimeraDenovo (with options method = "concensus"), separately for each study. The resulting chimera-filtered ASV table comprised 206,982,673 reads across 725,682 ASVs. Only ASVs matched by at least two reads (across all samples) were kept for downstream analyses in order to eliminate spurious sequences. Because we were mainly interested to check if the number of detected ASVs would be substantially (i.e., orders of magnitude) higher than the number of detected OTUs and because the DADA2 pipeline includes an algorithm for removing sequencing errors, we did not filter out ASVs found only in a single sample so as not risk underestimating the number of exact sequence variants. ASVs were taxonomically identified using SILVA and a consensus approach, as described above for OTUs, resulting in 580,965 prokaryotic ASVs, accounting for 181,673,137 reads across 5,584 samples. (Note that some samples did not pass the various filtering/merging steps.) A summary of AG samples, including sequence accession numbers, is provided as S3 Data.

To compare the number of ASVs and OTUs detected, we also analyzed the same set of quality-filtered reads as used for the above DADA2 analysis using our OTU-clustering approach utilized for the full GPC. Specifically, quality-filtered nonmerged paired-end reads, produced by the first step in the DADA2 pipeline, were used as input to the GPC clustering pipeline described above. This yielded 390,893 prokaryotic sequence clusters at 99% similarity accounting for 190,247,727 reads or 173,166 prokaryotic sequence clusters at 97% similarity accounting for 192,718,873 reads. For a comparison of ASVs and sequence clusters obtained for various numbers of studies included, see S13 Fig.

### Accumulation curves

Accumulation curves of OTUs discovered, as a function of studies included, were calculated as follows. For any given number of studies *N*, we randomly chose *N* studies in the GPC and counted the number of OTUs detected in at least one of the chosen studies. We repeated this step 100 independent times and averaged the number of OTUs counted each time. By performing this process for various *N* (from 1 to 492), we obtained the accumulation curves shown in Fig 1A and 1B.

### Estimating global OTU richness based on incidence frequencies

To estimate the total number of OTUs globally using the statistical estimators described in the main text (iChao2, ICE, CatchAll, breakaway, tWLRM), we considered each study as an independent sampling unit and counted the number of OTUs found in exactly one sampling unit (*Q*_1_), in exactly two sampling units (*Q*_2_), and so on. Note that since our last quality filter, by which we only kept OTUs found in at least two samples of the same study, was applied separately for each study, every study can indeed be considered as an independent sampling unit. Estimates and standard errors were either calculated using the R package breakaway (breakaway and tWLRM [48]), the R package SpadeR (iChao2 and ICE [105]), or the CatchAll software (CatchAll with 3-mixed exponential model [46]).

The assumption of the above estimators that sampling units are equivalent (e.g., of similar effort) is potentially violated by the GPC, since each included study was performed in a different environment and by using different techniques. To check whether our estimates are affected by this caveat, we also used a variant of iChao2 ("iChao2split"), whereby we randomly assigned studies to four complementary and equally sized groups and considered each group as a single independent global sampling unit. Hence, iChao2split considered the number of OTUs found in only one study group (*Q*_1_), in exactly two study groups (*Q*_2_), in three study groups (*Q*_3_), and in all four study groups (*Q*_4_). The splitting was randomly repeated 100 times, and the obtained estimates were averaged (Fig 1E); the standard error was set to the standard deviation of estimates across repeated splittings.

We mention that analogous estimators exist (e.g., "iChao1") for estimating richness in a community based on the observed OTU abundances (such as sequencing read counts) in a single reference sample [41]. Such abundance-based estimators are not suited for our data set for two reasons: first, to obtain a single globally ranging reference sample, we would need to pool all GPC samples so as to obtain a measure of abundance for the various OTUs. However, read counts from separate amplicon-sequencing samples cannot be combined to obtain a measure of global OTU abundances since the total number of cells that was present in each sample is unknown and sequencing depths varied between samples. Second, typical abundance-based estimators such as iChao1 rely on knowing the number of singleton OTUs (i.e., comprising only one read); however, singleton OTUs have a high probability of being spurious and can thus not be reliably used to estimate OTU richness [37]. In fact, singleton OTUs, as well as OTUs found in at most one sample, were omitted from the GPC to minimize spurious OTUs. Note that this filter corresponds to increasing the OTU detection threshold in each study, just as sequencing depth affects detection thresholds. Since the incidence-based richness estimators used in this study all account for finite (a priori unknown and potentially variable) detection probabilities, their applicability is not expected to be substantially compromised by a systematic application of this filter. This is roughly analogous to performing a mark-recapture–based assessment of wildlife population size; a systematic decrease of capturing effort may increase the variance of the resulting estimate, but it will not affect the expected value of that estimate.

### Estimating the fraction of cells represented by the GPC

To estimate the fraction of prokaryotic cells currently detectable by 16S amplicon sequencing that is represented by GPC OTUs (i.e., at 97% similarity in 16S), we calculated the probability (*P*) that a single additional read would hit a GPC OTU, as follows. Based on the number of OTUs with exactly two reads (*N*_2_ = 87,940) as well as the total number of reads (*N* = 1,734,042,763) and using the Good–Turing frequency formula [38], we estimate the total probability of hitting an OTU with one read in the GPC to be $P_{1}=2N_{2}/N=0.000101$. (Note that OTUs with one hit were omitted from the final GPC.) Using the fact that the total estimated probability of hitting an OTU with zero reads in the GPC (*P*_0_) is not greater than *P*_1_ (it is more probable to rehit some OTU with one read than to hit some OTU with zero reads) and the fact that $P\approx 1-(P_{0}+P_{1})$, we obtain the lower bound $P\geq 1-2P_{1}=99.98\%$. Hence, the probability of a single additional amplicon sequence hitting an OTU with ≥2 reads in the GPC is estimated to be P≥99.98%. An overview of computed probabilities for various clustering thresholds is given in S7 Table.

### Distribution of relative OTU abundances

To estimate the distribution of relative OTU abundances in the GPC, we proceeded as follows. First, for each OTU in the GPC, we estimated its relative abundance (*α*) in each sample based on the number of assigned reads and using the Good–Turing frequency estimator [38,106]:

$$\alpha =\frac{\left(r+1\right)}{N}\frac{N_{r+1}}{N_{r}},$$ (1)

in which *r* is the number of reads assigned to the OTU, *N*_*r*_ is the number of OTUs in the sample with exactly *r* reads, and *N* is the total number of reads in the sample. We note that the Good–Turing frequency estimator is widely used in biological statistics and has been repeatedly shown to be more robust than simply using the fraction of assigned reads [106,107]. Next, we averaged the relative abundances of each OTU across all samples to obtain its MRA in the GPC. We emphasize that we calculated MRAs separately for each sample, even though MRAs from shallower sequenced samples may be less accurate. This approach was preferred over the alternative of simply calculating the fraction of reads assigned to an OTU when all samples are pooled because samples differ drastically in sequencing depth; thus, OTUs that happen to occur in deeply sequenced samples would appear to be more abundant than OTUs in shallowly sequenced samples. Similarly, pooling within studies was also avoided because sequencing depth varied widely even among samples of the same study, and samples were usually not technical replicates; hence, MRAs calculated for a given study (after pooling) would be biased toward organisms that happened to be present in deeply sequenced samples. By calculating MRAs separately for each sample prior to averaging, we avoid biases toward OTUs in more deeply sequenced samples.

Next, we grouped OTUs into small, equally sized MRA intervals (on a logarithmic scale) to calculate a frequency histogram of MRAs in the GPC. We note that the resulting frequency histogram should not be interpreted as a true OTU abundance distribution because it only includes OTUs discovered by the GPC and may thus be artificially positively skewed [108]. To estimate the probability that an extant OTU in an MRA interval was included in the GPC (*P*(*α*), in which *α* is the center of the MRA interval) and, from that, the total number of extant OTUs in each MRA interval, we proceeded as follows. We randomly removed half of the quality- and chimera-filtered reads and repeated the OTU clustering and analyses described above, thus obtaining a rarefied variant of the GPC (rGPC). A total of 514,432 high-fidelity prokaryotic OTUs were retrieved from the rGPC. We then calculated the frequency histogram of MRAs for the rGPC and compared it to the one obtained from the GPC to estimate *P*(*α*) for each MRA interval. Specifically, we assumed that the number of reads assigned to an OTU in any given MRA interval was Poisson-distributed and that the probability of being discovered was given by the probability of being matched by at least two reads, i.e.,

$$P(\alpha )=1-e^{-\lambda (\alpha )}-\lambda (\alpha )e^{-\lambda (\alpha )},$$ (2)

in which *λ*(*α*) is the unknown rate of the Poisson distribution for that MRA interval. Since the rGPC includes half the reads of the GPC, the probability of OTU discovery by the rGPC is $P_{r}(\alpha )=1-e^{-\lambda_{r}(\alpha )}-\lambda_{r}(\alpha )e^{-\lambda_{r}(\alpha )}$, in which *λ*_*r*_ *= λ*/2. For each MRA interval, we estimated *λ*(*α*) by numerically solving the equation

$$\frac{f(\alpha )}{f_{r}(\alpha )}=\frac{1-e^{-\lambda (\alpha )}-\lambda (\alpha )e^{-\lambda (\alpha )}}{1-e^{-\frac{1}{2}\lambda (\alpha )}-\frac{1}{2}\lambda (\alpha )e^{-\frac{1}{2}\lambda (\alpha )}},$$ (3)

in which *f*(*α*) and *f*_*r*_(*α*) is the number of OTUs in the focal MRA interval, discovered by the GPC and the rGPC, respectively. From the estimated *λ*(*α*), we thus obtained *P*(*α*) via Eq 2 and the total number of extant OTUs in the MRA interval as F(α)=f(α)/P(α).

Following suggestions by Shoemaker and colleagues [11], who concluded that microbial communities are often well described by log-normal species abundance distributions, a log-normal model was fitted to the reconstructed OTU MRA distribution *F*:

$$F(\alpha )∼\frac{S}{\sqrt{2\pi \sigma^{2}}}\exp\left[-\frac{{\left(\log(\alpha )-\mu \right)}^{2}}{2\sigma^{2}}\right],$$ (4)

in which *μ*, *σ*, and *S* are fitted parameters. Fitting was performed via least-squares. The fitted log-normal model was integrated over the entire real axis to obtain an estimate for the total number of extant prokaryotic OTUs.

## Supporting information

S1 Data Sample summary and accession numbers. (TSV)

S2 Data OTU incidence frequency tables. OTU, operational taxonomic unit. (ZIP)

S3 Data Sample summary and accession numbers for AG subset. (TSV)

S1 Text The pitfalls of extrapolating host-specific microbial diversity estimates. (PDF)

S2 Text An upper bound for the number of extant OTUs at steady state. OTU, operational taxonomic unit.
(PDF)

S1 Fig Sample locations. Geographical locations of GPC samples (A) and the subset of GPC samples included in the DADA2 comparison (B). Only samples with publicly accessioned latitude and longitude information are shown (25,796 samples in A; 4,860 samples in B). DADA2; GPC, Global Prokaryotic Census.
(JPG)

S2 Fig Samples and studies per OTU. Frequency histograms of the number of samples (top row) and the number of studies (bottom row) in which each GPC OTU was found in for bacteria (left column) and archaea (right column). Only OTUs found in at least two samples of the same study are included in the GPC so as to avoid spurious OTUs. In A and B, the left-most bar refers to a number of samples equal to two. GPC, Global Prokaryotic Census; OTU, operational taxonomic unit.
(PDF)

S3 Fig Distribution of mean relative cluster abundances (99%, 95%, and 90% similarities). Frequency histogram of MRAs of prokaryotic 16S clusters (A: 99% similarity, B: 95% similarity, C: 90% similarity) discovered by the GPC (grey continuous line), of clusters discovered by the rGPC (grey dashed lines), and of all extant clusters as estimated using a probabilistic model of OTU discovery (blue continuous curve). The probabilistic model was fitted separately for each MRA interval by comparing the discovery rates of the GPC and the rGPC. The blue dashed curve shows a log-normal distribution model fitted to the estimated MRA distribution of extant clusters (*R*^2^ > 0.96 in all cases). GPC, Global Prokaryotic Census; MRA, mean relative abundance; OTU, operational taxonomic unit; rGPC, rarefied variant of the GPC.
(PDF)

S4 Fig Collector's curves of clusters versus studies (at 99% similarity). Accumulation curves, showing the number of bacterial (A) and archaeal (B) clusters (99% similarity in the 16S-V4 region) discovered, depending on the number of distinct studies included. Curves are averaged over 100 random subsamplings, and whiskers show corresponding standard deviations. Continuous curves were calculated using all studies (worldwide), while blue dashed curves were calculated using solely studies performed in the Americas or near American coasts.
(PDF)

S5 Fig Prokaryotic richness estimates (Americas versus globally). Prokaryotic 16S cluster richness at 97% (A) and 99% (B) clustering similarity, estimated using various statistical estimators and based on studies from the Americas (blue bars) or an equal number of studies chosen randomly from the global data set (grey bars). The number of OTUs discovered by the GPC is included for comparison (last bar). Error bars indicate standard errors, estimated from the underlying models; most standard errors are likely underestimated by the models, so the variability between models is probably a more honest assessment of uncertainty. GPC, Global Prokaryotic Census; OTU, operational taxonomic unit.
(PDF)

S6 Fig Richness of phyla and coverage by SILVA and the GPC (at 97% similarity). (A) Estimated number of OTUs (97% similarity in the 16S-V4 region) globally, within various prokaryotic phyla. Estimated based on the coverage of SILVA by the GPC (subfigure C) and the number of OTUs in the GPC. Only phyla including at least 10 entries in SILVA (release 132, set NR99) [14] and estimated to contain at least 10 extant OTUs are considered. (B) Fraction of GPC OTUs that could be mapped to SILVA NR99 at similarity ≥97%, as a proxy for global OTU richness covered by SILVA, within the same phyla as in A. (C) Fraction of SILVA NR99 sequences that could be mapped to the GPC at similarity ≥97%, as a proxy for global OTU richness covered by the GPC, within the same phyla as in A. GPC, Global Prokaryotic Census; NR, nonredundant; OTU, operational taxonomic unit; SILVA.
(PDF)

S7 Fig Richness of phyla and coverage by SILVA and the GPC (at 99% similarity). (A) Estimated number of 16S sequence clusters (99% similarity in the 16S-V4 region) globally, within various prokaryotic phyla. Estimated based on the coverage of SILVA by the GPC (subfigure C) and the number of clusters in the GPC. Only phyla including at least 10 entries in SILVA (release 132, set NR99) and estimated to contain at least 10 extant clusters are shown. (B) Fraction of GPC clusters that could be mapped to SILVA NR99 at similarity ≥99%, as a proxy for global OTU richness covered by SILVA, within the same phyla as in A. (C) Fraction of SILVA NR99 sequences that could be mapped to GPC clusters at similarity ≥99%, as a proxy for global cluster richness covered by the GPC, within the same phyla as in A. GPC, Global Prokaryotic Census; NR, nonredundant; OTU, operational taxonomic unit; SILVA.
(PDF)

S8 Fig Richness of phyla and coverage by SILVA and the GPC (at 95% similarity). (A) Estimated number of 16S sequence clusters (95% similarity in the 16S-V4 region) globally, within various prokaryotic phyla. Estimated based on the coverage of SILVA by the GPC (subfigure C) and the number of clusters in the GPC. Only phyla including at least 10 entries in SILVA (release 132, set NR99) and estimated to contain at least 10 extant clusters are shown. (B) Fraction of GPC clusters that could be mapped to SILVA NR99 at similarity ≥95%, as a proxy for global cluster richness covered by SILVA, within the same phyla as in A. (C) Fraction of SILVA NR99 sequences that could be mapped to GPC clusters at similarity ≥95%, as a proxy for global cluster richness covered by the GPC, within the same phyla as in A. GPC, Global Prokaryotic Census; NR, nonredundant; SILVA.
(PDF)

S9 Fig Richness of phyla and coverage by SILVA and the GPC (at 90% similarity). (A) Estimated number of 16S sequence clusters (90% similarity in the 16S-V4 region) globally, within various prokaryotic phyla. Estimated based on the coverage of SILVA by the GPC (subfigure C) and the number of clusters in the GPC. Only phyla including at least 10 entries in SILVA (release 132, set NR99) and estimated to contain at least 10 extant clusters are shown. (B) Fraction of GPC clusters that could be mapped to SILVA NR99 at similarity ≥90%, as a proxy for global cluster richness covered by SILVA, within the same phyla as in A. (C) Fraction of SILVA NR99 sequences that could be mapped to GPC clusters at similarity ≥90%, as a proxy for global cluster richness covered by the GPC, within the same phyla as in A. GPC, Global Prokaryotic Census; NR, nonredundant; SILVA.
(PDF)

S10 Fig Richness of classes and coverage by SILVA and the GPC (at 99% similarity). (A) Estimated number of 16S sequence clusters (99% similarity in the 16S-V4 region) globally, within various prokaryotic classes. Estimated based on the coverage of SILVA by the GPC (subfigure C) and the number of clusters in the GPC. Only classes including at least 10 entries in SILVA (release 132, set NR99) and estimated to contain at least 10 extant clusters are shown. (B) Fraction of GPC clusters that could be mapped to SILVA NR99 at similarity ≥99%, as a proxy for global cluster richness covered by SILVA, within the same classes as in A. (C) Fraction of SILVA NR99 sequences that could be mapped to GPC clusters at similarity ≥99%, as a proxy for global cluster richness covered by the GPC, within the same classes as in A. GPC, Global Prokaryotic Census; NR, nonredundant; SILVA.
(PDF)

S11 Fig Richness of classes and coverage by SILVA and the GPC (at 97% similarity). (A) Estimated number of 16S sequence clusters (97% similarity in the 16S-V4 region) globally, within various prokaryotic classes. Estimated based on the coverage of SILVA by the GPC (subfigure C) and the number of clusters in the GPC. Only classes including at least 10 entries in SILVA (release 132, set NR99) and estimated to contain at least 10 extant clusters are shown. (B) Fraction of GPC clusters that could be mapped to SILVA NR99 at similarity ≥97%, as a proxy for global OTU richness covered by SILVA, within the same classes as in A. (C) Fraction of SILVA NR99 sequences that could be mapped to GPC clusters at similarity ≥97%, as a proxy for global cluster richness covered by the GPC, within the same classes as in A. GPC, Global Prokaryotic Census; NR, nonredundant; OTU, operational taxonomic unit; SILVA.
(PDF)

S12 Fig Richness of classes and coverage by SILVA and the GPC (at 95% similarity). (A) Estimated number of 16S sequence clusters (95% similarity in the 16S-V4 region) globally, within various prokaryotic classes. Estimated based on the coverage of SILVA by the GPC (subfigure C) and the number of clusters in the GPC. Only classes including at least 10 entries in SILVA (release 132, set NR99) and estimated to contain at least 10 extant clusters are shown. (B) Fraction of GPC clusters that could be mapped to SILVA NR99 at similarity ≥95%, as a proxy for global cluster richness covered by SILVA, within the same classes as in A. (C) Fraction of SILVA NR99 sequences that could be mapped to GPC clusters at similarity ≥95%, as a proxy for global cluster richness covered by the GPC, within the same classes as in A. GPC, Global Prokaryotic Census; NR, nonredundant; SILVA.
(PDF)

S13 Fig Sequence clusters discovered at 97%, 99%, and 100% similarities (GPC subset AG). Number of prokaryotic 16S-V4 sequence clusters discovered at various similarity thresholds (97%, 99%, and 100%) and for various numbers of studies included. Only a subset of 111 studies were used in this analysis (subset "AG"). Clusters at 97% and 99% were generated using cd-hit-otu, as described in the main article; clusters at 100% correspond to exact ASVs, generated using DADA2. ASV, amplicon sequence variant; DADA2; GPC, Global Prokaryotic Census.
(PDF)

S14 Fig Distances of GPC sequence clusters to SILVA. (A) Fraction of GPC 16S-V4 sequences clusters (clustered at 97% identity) that could be matched to a SILVA entry at various similarity thresholds (fraction of nucleotide mismatches). Note the jump at around 50% similarity. (B) Distribution of distances between GPC 16S-V4 sequence clusters (clustered at 97% identity) and SILVA (measured as percent of nucleotide mismatches to the closest match in SILVA). Note the sharp peak within the distance interval of 40%–50%. Figures A and B contain the same information, shown in alternative ways. Note that the horizontal axis shows similarities in A and distances in B. (C, D) Same as A and B but for GPC sequence clusters at 99% identity. Observe that the peak in the 40%–50% distance interval is much smaller for 99%-clusters than for 97%-clusters. Also see S15 Fig for a comparison with exact amplicon sequence variants. GPC, Global Prokaryotic Census; SILVA.
(PDF)

S15 Fig Distances of GPC sequence clusters and ASVs to SILVA (GPC subset AG). (A) Distribution of distances between 16S-V4 sequence clusters (clustered at 97% identity) and SILVA (measured as percent of nucleotide mismatches to the closest match in SILVA). Clusters were generated from a subset of 111 studies (subset "AG"). Note the peak within the distance interval 40%–50% and the absence of any OTUs to the right of that peak. (B) Similar to A but for sequence clusters at 99% identity. (C) Similar to A but for exact ASVs generated using DADA2. Observe that the peak in the 40%–50% distance interval is much smaller for 99%-clusters than for 97%-clusters and almost disappears in the case of ASVs. ASV, amplicon sequence variant; DADA2; GPC, Global Prokaryotic Census; OTU, operational taxonomic unit; SILVA.
(PDF)

S1 Table Estimated numbers of extant prokaryotic 16S clusters worldwide. Number of extant prokaryotic 16S sequence clusters (at 90%, 95%, 97%, or 99% similarities in the 16S-V4 region), estimated using various methods, including iChao2, iChao2split, ICE, CatchAll, breakaway, tWLRM, based on the coverage of SILVA, or the RDP (see main text for details), and based on a log-normal model fitted to OTU MRAs. Uncertainties (±) correspond to standard errors, wherever applicable. The last row lists the number of clusters discovered by the GPC. NA indicates that the estimator did not converge. GPC, Global Prokaryotic Census; ICE, incidence coverage-based estimator; MRA, mean relative abundance; NA, not available; OTU, operational taxonomic unit; RDP, Ribosomal Database Project; SILVA; tWLRM, transformed weighted linear regression model.
(PDF)

S2 Table Recapture fractions of other data sets by the GPC (at 97% similarity). Fraction of recaptured (at 97% similarity) prokaryotic 16S sequences in third party data sets, including the EMP, the SILVA (NR99) database release 132, 16S sequences assembled from metagenomes (UBA), bacterial 16S sequences extracted from IMG/M metagenomes, the RDP release 11, and the Genome Taxonomic Database release 86.1, by GPC OTUs. In cases in which the number of OTUs in the third party data set was low (<1,000), the numbers of OTUs compared are indicated in brackets. EMP, Earth Microbiome Project; GPC, Global Prokaryotic Census; IMG/M, Integrated Microbial Genomes and Microbiomes; NR, nonredundant; OTU, operational taxonomic unit; RDP, Ribosomal Database Project; SILVA; UBA, Uncultivated Bacteria and Archaea.
(PDF)

S3 Table Recapture fractions of other data sets by the GPC (at 99% similarity). Fraction of recaptured (at ≥99% similarity) prokaryotic 16S sequences in third party data sets, including the EMP, the SILVA (NR99) database release 132, 16S sequences assembled from metagenomes (UBA), bacterial 16S sequences extracted from IMG/M metagenomes, and the RDP release 11, by 16S sequence clusters (99% similarity) in the GPC. EMP, Earth Microbiome Project; GPC, Global Prokaryotic Census; IMG/M, Integrated Microbial Genomes and Microbiomes; NR, nonredundant; RDP, Ribosomal Database Project; SILVA; UBA, Uncultivated Bacteria and Archaea.
(PDF)

S4 Table Recapture fractions of other data sets by the GPC (at 95% similarity). Fraction of recaptured (at ≥95% similarity) prokaryotic 16S sequences in third party data sets, including the EMP, the SILVA (NR99) database release 132, 16S sequences assembled from metagenomes (UBA), bacterial 16S sequences extracted from IMG/M metagenomes, and the RDP release 11, by 16S sequence clusters (95% similarity) in the GPC. EMP, Earth Microbiome Project; GPC, Global Prokaryotic Census; IMG/M, Integrated Microbial Genomes and Microbiomes; NR, nonredundant; RDP, Ribosomal Database Project; SILVA; UBA, Uncultivated Bacteria and Archaea.
(PDF)

S5 Table Recapture fractions of other data sets by the GPC (at 90% similarity). Fraction of recaptured (at ≥90% similarity) prokaryotic 16S sequences in third party data sets, including the EMP, the SILVA (NR99) database release 132, 16S sequences assembled from metagenomes (UBA), bacterial 16S sequences extracted from IMG/M metagenomes, and the RDP release 11, by 16S sequence clusters (90% similarity) in the GPC. EMP, Earth Microbiome Project; GPC, Global Prokaryotic Census; IMG/M, Integrated Microbial Genomes and Microbiomes; NR, nonredundant; RDP, Ribosomal Database Project; SILVA; UBA, Uncultivated Bacteria and Archaea.
(PDF)

S6 Table Prokaryotic richness contained in SILVA. Number of 16S clusters in SILVA release 132 within various taxa, obtained when clustering only the V4 region (200 bp, starting at *E*. *coli* position 516) or the full 16S gene (approximately 1,500 bp) at 97% or 99% similarity. SILVA.
(PDF)

S7 Table Estimated numbers of living prokaryotic cells represented by the GPC (at 90%, 95%, 97%, or 99% similarities). Number of 16S sequence clusters in the GPC with exactly two reads (*N*_2_) and probability that a single additional amplicon sequence would hit a GPC cluster (*P*, estimated using the Good–Turing frequency formula, see Methods for details) for various clustering similarities. GPC, Global Prokaryotic Census.
(PDF)

## Abbreviations

ASV: amplicon sequence variant
DADA2: Divisive Amplicon Denoising Algorithm
EMP: Earth Microbiome Project
GPC: Global Prokaryotic Census
GTDB: Genome Taxonomic Database
ICE: incidence coverage-based estimator
IMG/M: Integrated Microbial Genomes and Microbiomes
MRA: mean relative abundance
NR: nonredundant
OTU: operational taxonomic unit
RDP: Ribosomal Database Project
rGPC: rarefied variant of the GPC
tWLRM: transformed weighted linear regression model
UBA: Uncultivated Bacteria and Archaea
