# Supplementary material for: A census-based estimate of Earth's bacterial and archaeal diversity
Source: PLoS Biol. 2019 Feb 4;17(2):e3000106. doi: 10.1371/journal.pbio.3000106 (PMC6361415; doi:10.1371/journal.pbio.3000106)
Supplement: S1 Text — (PDF) [file pbio.3000106.s004.pdf]

A census-based estimate of Earth's bacterial and archaeal diversity - S1 Text -

Stilianos Louca[1,2,3,4], Florent Mazel[3,5], Michael Doebeli[3,4,6] & Laura Wegener Parfrey[3,4,5]

[1]*Department of Biology, University of Oregon, Eugene, Oregon, USA*
[2]*Institute of Ecology and Evolution, University of Oregon, Eugene, Oregon, USA*
[3]*Biodiversity Research Centre, University of British Columbia, Vancouver, Canada*
[4]*Department of Zoology, University of British Columbia, Vancouver, Canada*
[5]*Department of Botany, University of British Columbia, Vancouver, Canada*
[6]*Department of Mathematics, University of British Columbia, Vancouver, Canada*

The pitfalls of extrapolating host-specific microbial diversity estimates

Estimating global species diversity has long been a challenging endeavor, with microorganisms constituting a particularly cryptic aspect of Life [1, 2]. In a recent meta-analysis, Larsen et al. [3] attempted to estimate the global number of host-associated bacterial operational taxonomic units (OTUs, a bacterial species analog), by extrapolating an estimated number of 10.7 unique OTUs per insect species to an estimated 163.2 million animal species worldwide, concluding that there exist billions of bacterial OTUs ($10.7 \times 163.2 \times 10^6$). Their findings suggested the existence of an immense undiscovered bacterial diversity and triggered anew discussions about the relative contribution of microbial taxa to the "Pie of Life" [3]. Here we explain why Larsen et al.'s extrapolation is mathematically erroneous and likely led to a severe overestimate of global host-associated bacterial OTU diversity, henceforth denoted N.

Based on pairwise comparisons of congeneric insect species, Larsen et al. calculated the average number of OTUs per host species and the average number of OTUs unique to each host species when compared to another randomly chosen congeneric species. For example, using previously published data [4], Larsen et al. calculated that each species in the *Cephalotes* genus (turtle ants) has on average 19 bacterial OTUs and that on average 9 OTUs were shared in any given comparison of two *Cephalotes* species, concluding that each *Cephalotes* species has 10 unique bacterial OTUs. Larsen et al. obtained comparable results for the genera *Drosophila* and *Nasonia*, and thus concluded that each insect species has on average $U = 10.7$ unique bacterial OTUs, and hence that $S = 163.2 \times 10^6$ animal species must host $\sim S \times U$ distinct OTUs. Larsen et al.'s assumption that U generally applies to animal species other than insects is itself questionable, and so is their relatively high estimate of 163 million animal species (e.g., Mora et al. [5] predicts only $\sim$7.7 million animal species). Nevertheless, here we shall focus on a more fundamental (mathematical) issue in Larsen et al.'s reasoning, by showing that the product $S \times U$ is under no circumstances a proper estimator for N, for two important reasons.

First, Larsen et al. estimated U solely by comparing congeneric host species, and it is known that substantial overlap exists between the microbiota of different host genera and even of distantly related animal taxa. This overlap is partly due to host trait convergences [6–9], and partly due to the fact that some bacteria found

in hosts are not host-specific but merely taken up temporarily from the environment. For example, the gut microbiota of fish overlap substantially with those of mammals and insects [7]. The extent of such overlaps cannot possibly be inferred solely from congeneric host species comparisons, but it is needed for correctly extrapolating to all animal taxa. To illustrate our point, in the (admittedly extreme) hypothetical scenario where each animal family has the same set of bacterial OTUs, N would be equal to the OTU diversity within a single animal family; purely congeneric host species comparisons could never rule out such a scenario.

Second, even if there was no overlap between the microbiota of distinct animal genera (an unrealistic scenario, which would result in the highest possible N, given Larsen et al.'s U and S), the proper estimate for N would be $G \times N_{\mathrm{g}}$, where G is the number of animal genera and N_{g} is the number of bacterial OTUs per animal genus. Assuming that Larsen et al.'s pairwise congeneric host species comparisons are indeed representative, N_{g} may be estimated for a particular animal genus using the Chao2 incidence-based diversity estimator [10, Eq. 11a]. The Chao2 estimator was originally designed for estimating total (observed + unobserved) OTU diversity in a "region" or "community" (here, an animal genus) based on the number of OTUs observed once or twice in a set of independent "sampling units" (here, two congeneric host species), and is thus particularly suited for interpreting Larsen et al.'s data. In the latter case, the Chao2 estimator takes the simple form

$$N_{\mathrm{g}} = \frac{B^2}{B - U}, \tag{1}$$

where B is the average number of bacterial OTUs found in a single host species. Note that the above estimator is mathematically analogous to mark-recapture approaches conventionally used to estimate the size of a population [11], with B being analogous to the number of individuals marked in the first survey and $B - U$ being analogous to the number of marked individuals recaptured in a subsequent survey.

Taking Larsen et al.'s calculations for *Cephalotes* as an example ($B = 19, U = 10$), Eq. (1) would predict only $N_{\mathrm{g}} = 40.1$ OTUs for the entire *Cephalotes* genus. We point out that an approach analogous to Larsen et al. [3], i.e. estimating N_{g} as $S_{\mathrm{g}} \times U$ (where S_{g} is the number of *Cephalotes* species), would fail even for a single genus. The reason is that the number of OTUs unique to a host species, when compared to a single random congeneric species, is generally greater than the number of OTUs unique to a host species when compared to all other congeneric species together. In other words, the number of OTUs that are truly unique to a host species (i.e., not found in any other host), is generally much smaller than the average number of OTUs unique to a host in pairwise comparisons. The *Cephalotes* genus contains about 130 described species [3], and Mora et al. [5, Fig. S4] also estimate that there are about 100 species per animal genus. Assuming $\sim$100 species per animal genus, and assuming that Larsen et al.'s estimate of global animal species diversity is correct, there are $G \sim S/100 \approx 1,632,000$ animal genera, and hence at most 65,443,200 bacterial OTUs globally $(1,632,000 \times 40.1)$.

In conclusion, even if Larsen et al.'s estimates of U and B for insect genera can be generalized to all animal genera, and even if there was no overlap between the microbiota of distinct animal genera (evidently a strongly unrealistic scenario [6, 7]), and even if Larsen et al.'s unusually high estimate of $S = 163.2 \times 10^6$ animal species was accurate, a mathematically correct use of Larsen et al.'s U, B and S would predict a global bacterial diversity 25 times lower than claimed by Larsen et al. [3]. Using the animal diversity estimate by Mora et al. [5], the estimate further reduces to 3,087,700 bacterial OTUs. Taking into account the substantial overlap between animal genera would further reduce the estimated N. For example, even at a conservative overlap of only 0.1% between any two randomly chosen genera, the global host-associated bacterial diversity estimate would drop to 40,100 OTUs (applying the Chao2 estimator for $B = 40.1$ and $U = 0.999 \cdot B$).

References

1 Whitman WB, Coleman DC, Wiebe WJ. Prokaryotes: The unseen majority. Proc Natl Acad Sci USA. 1998; 95(12):6578–6583.

2 Hug LA, Baker BJ, Anantharaman K, Brown CT, Probst AJ, Castelle CJ, et al. A new view of the tree of life. Nat Microbiol. 2016; 1:16048 EP. doi:https://doi.org/10.1038/nmicrobiol.2016.48.

3 Larsen BB, Miller EC, Rhodes MK, Wiens JJ. Inordinate fondness multiplied and redistributed: the number of species on Earth and the new pie of life. Q Rev Biol. 2017; 92(3):229–265. doi:https://doi.org/10.1086/693564.

4 Sanders JG, Powell S, Kronauer DJC, Vasconcelos HL, Frederickson ME, Pierce NE. Stability and phylogenetic correlation in gut microbiota: lessons from ants and apes. Mol Ecol. 2014; 23(6):1268–1283. doi:https://doi.org/10.1111/mec.12611.

5 Mora C, Tittensor DP, Adl S, Simpson AG, Worm B. How many species are there on Earth and in the ocean? PLoS Biol. 2011; 9(8):e1001127.

6 Muegge BD, Kuczynski J, Knights D, Clemente JC, González A, Fontana L, et al. Diet drives convergence in gut microbiome functions across mammalian phylogeny and within humans. Science. 2011; 332(6032):970–974.

7 Sullam KE, Essinger SD, Lozupone CA, O'connor MP, Rosen GL, Knight R, et al. Environmental and ecological factors that shape the gut bacterial communities of fish: a meta-analysis. Mol Ecol. 2012; 21(13):3363–3378. doi:https://doi.org/10.1111/j.1365-294X.2012.05552.x.

8 Aylward FO, Suen G, Biedermann PHW, Adams AS, Scott JJ, Malfatti SA, et al. Convergent bacterial microbiotas in the fungal agricultural systems of insects. mBio. 2014; 5(6):e02077–14. doi:https://doi.org/10.1128/mBio.02077-14.

9 Salzman S, Whitaker M, Pierce NE. Cycad-feeding insects share a core gut microbiome. Biol J Linn Soc. 2018; p. bly017. doi:https://doi.org/10.1093/biolinnean/bly017.

10 Chao A, Chiu CH. Species richness: estimation and comparison. Wiley StatsRef: Statistics Reference Online. 2016; .

11 Krebs CJ. Ecological Methodology. Life Sciences. San Francisco, USA: Benjamin/Cummings; 1999.
