# Supplementary material for: A census-based estimate of Earth's bacterial and archaeal diversity
Source: PLoS Biol. 2019 Feb 4;17(2):e3000106. doi: 10.1371/journal.pbio.3000106 (PMC6361415; doi:10.1371/journal.pbio.3000106)
Supplement: S2 Text — OTU, operational taxonomic unit. (PDF) [file pbio.3000106.s005.pdf]

A census-based estimate of Earth's bacterial and archaeal diversity - S2 Text -

Stilianos Louca[1,2,3,4], Florent Mazel[3,5], Michael Doebeli[3,4,6] & Laura Wegener Parfrey[3,4,5]

[1]*Department of Biology, University of Oregon, Eugene, Oregon, USA*
[2]*Institute of Ecology and Evolution, University of Oregon, Eugene, Oregon, USA*
[3]*Biodiversity Research Centre, University of British Columbia, Vancouver, Canada*
[4]*Department of Zoology, University of British Columbia, Vancouver, Canada*
[5]*Department of Botany, University of British Columbia, Vancouver, Canada*
[6]*Department of Mathematics, University of British Columbia, Vancouver, Canada*

An upper bound for the number of extant OTUs at steady state

In the following we derive an expectation for the number of OTUs (N_o) expected under steady state conditions, i.e. assuming that global prokaryotic cell counts (N) are fairly constant and subject to random (phylogenetically uncorrelated) turnover at some constant rate (which is both the average per-capita birth rate as well as the average per-capita death rate, measured in generations per time). Simple cladogenic models predict that under these conditions the number of extant lineages (where each cell represents a distinct new lineage) that can be traced back to any past time point (known as "lineages through time") is given by

$$\tilde{N}(n) = \frac{N}{1+n}, \tag{1}$$

where n is the expected number of generations from that time point (per-capita birth rate $\times$ time) [1, 2]. Assuming that the 16S rRNA gene evolves neutrally at some constant drift rate r (measured in substitutions per site per generation), an OTU (defined by a divergence threshold of 3%) corresponds to a set of organisms (clade) that diverged $n \sim 0.3/(2r)$ generations ago. Hence, the expected number of OTUs is approximately $N_o = \tilde{N}(n) \approx 2rN/0.3$. Assuming $r = 4 \times 10^{-9}$–5×10^{-9} (based on mutation rates estimated for various bacterial loci [3, 4]) and assuming $N = 10^{30}$ cells [5], one obtains the estimate $N_o \sim 3.3 \times 10^{22}$–$2.7 \times 10^{23}$. Note that in reality prokaryotic cell deaths are not phylogenetically uncorrelated, i.e. closely related organisms (e.g. sister cells) are more likely to die together or survive together, because they are usually found in similar locations and exhibit similar traits. Such phylogenetic correlation in birth/death events would lead to a higher probability of older clades (e.g. at the OTU level) becoming extinct, and hence N_o is only an upper bound for the number of extant OTUs. In fact, our finding of much smaller global OTU diversity suggests that phylogenetically correlated cell turnover is a major driver in prokaryotic ecology and evolution.

References

1 Kendall DG. On some modes of population growth leading to RA Fisher's logarithmic series distribution. Biometrika. 1948; 35:6–15.

2 Nee S, May RM, Harvey PH. The reconstructed evolutionary process. Philos Trans R Soc Lond B Biol Sci. 1994; 344(1309):305–311.

3 Ochman H. Neutral Mutations and Neutral Substitutions in Bacterial Genomes. Mol Biol Evol. 2003; 20(12):2091–2096. doi:https://doi.org/10.1093/molbev/msg229.

4 Moran NA, McLaughlin HJ, Sorek R. The dynamics and time scale of ongoing genomic erosion in symbiotic bacteria. Science. 2009; 323(5912):379–382. doi:https://doi.org/10.1126/science.1167140.

5 Whitman WB, Coleman DC, Wiebe WJ. Prokaryotes: The unseen majority. Proc Natl Acad Sci USA. 1998; 95(12):6578–6583.
