# Supplementary figures and images for: A census-based estimate of Earth's bacterial and archaeal diversity
Source: PLoS Biol. 2019 Feb 4;17(2):e3000106. doi: 10.1371/journal.pbio.3000106 (PMC6361415; doi:10.1371/journal.pbio.3000106)

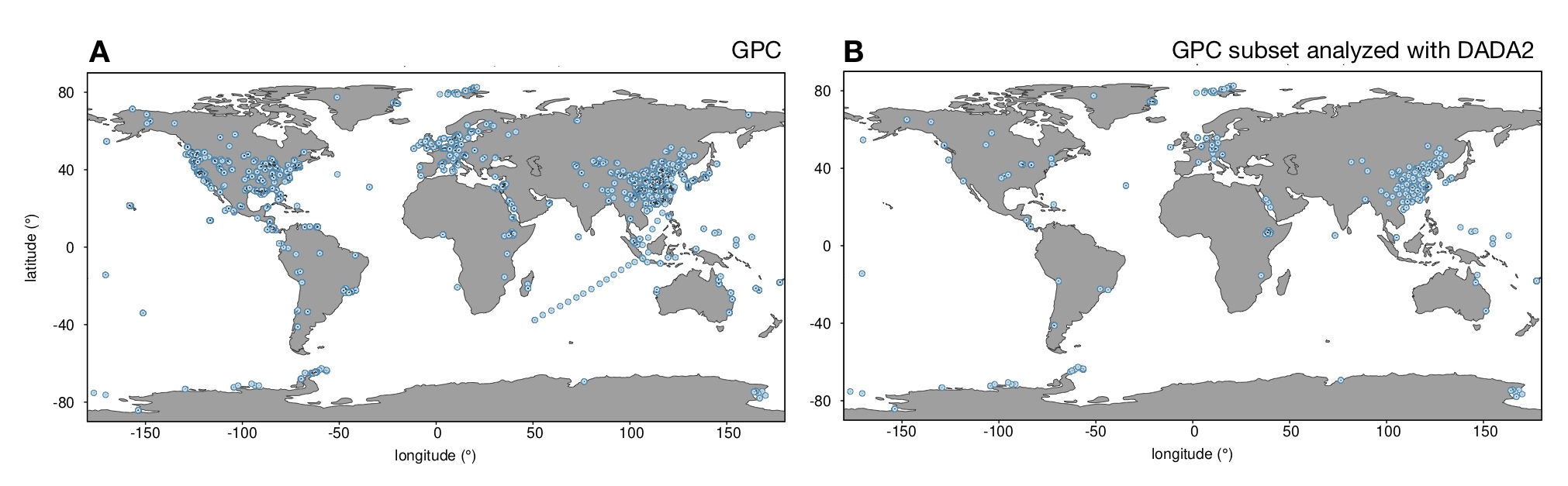

Supplement: S1 Fig — Geographical locations of GPC samples (A) and the subset of GPC samples included in the DADA2 comparison (B). Only samples with publicly accessioned latitude and longitude information are shown (25,796 samples in A; 4,860 samples in B). DADA2; GPC, Global Prokaryotic Census. (JPG) [file pbio.3000106.s006.jpg]

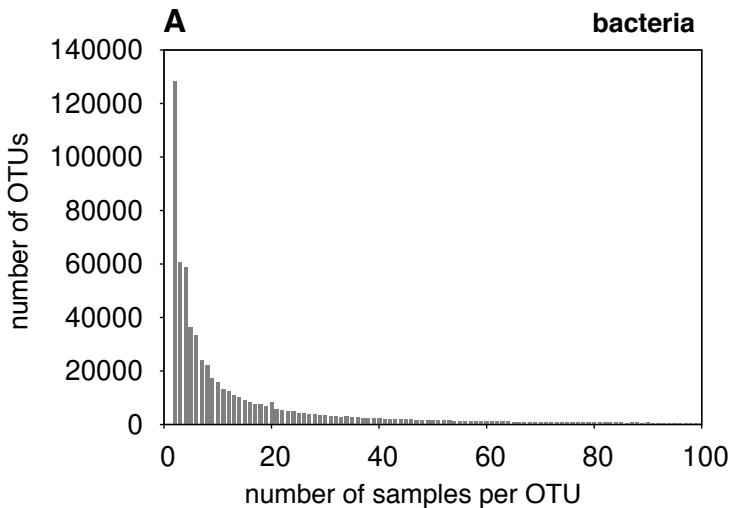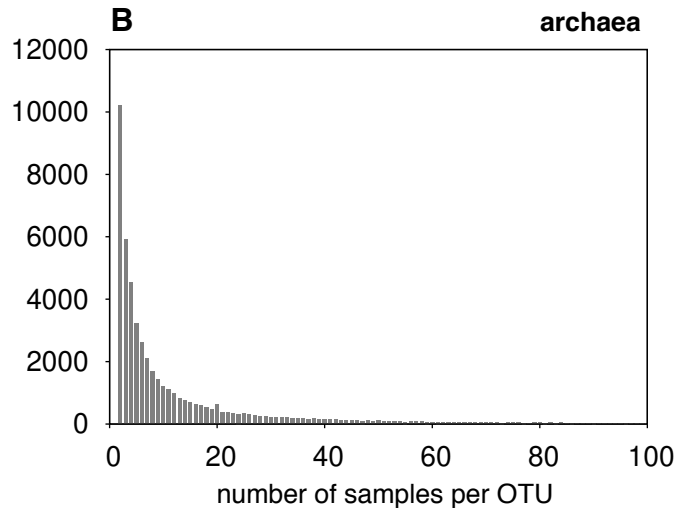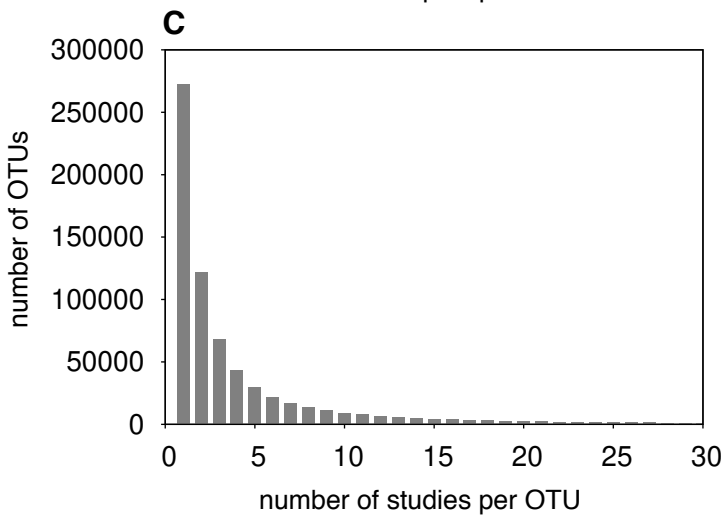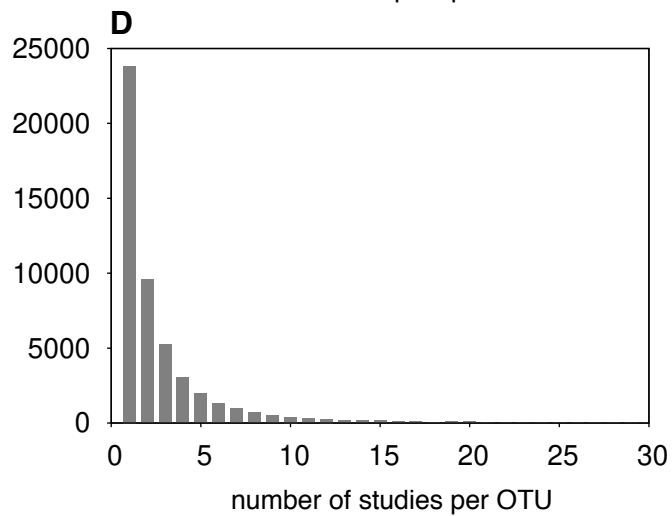

Supplement: S2 Fig — Frequency histograms of the number of samples (top row) and the number of studies (bottom row) in which each GPC OTU was found in for bacteria (left column) and archaea (right column). Only OTUs found in at least two samples of the same study are included in the GPC so as to avoid spurious OTUs. In A and B, the left-most bar refers to a number of samples equal to two. GPC, Global Prokaryotic Census; OTU, operational taxonomic unit. (PDF) [file pbio.3000106.s007.pdf]

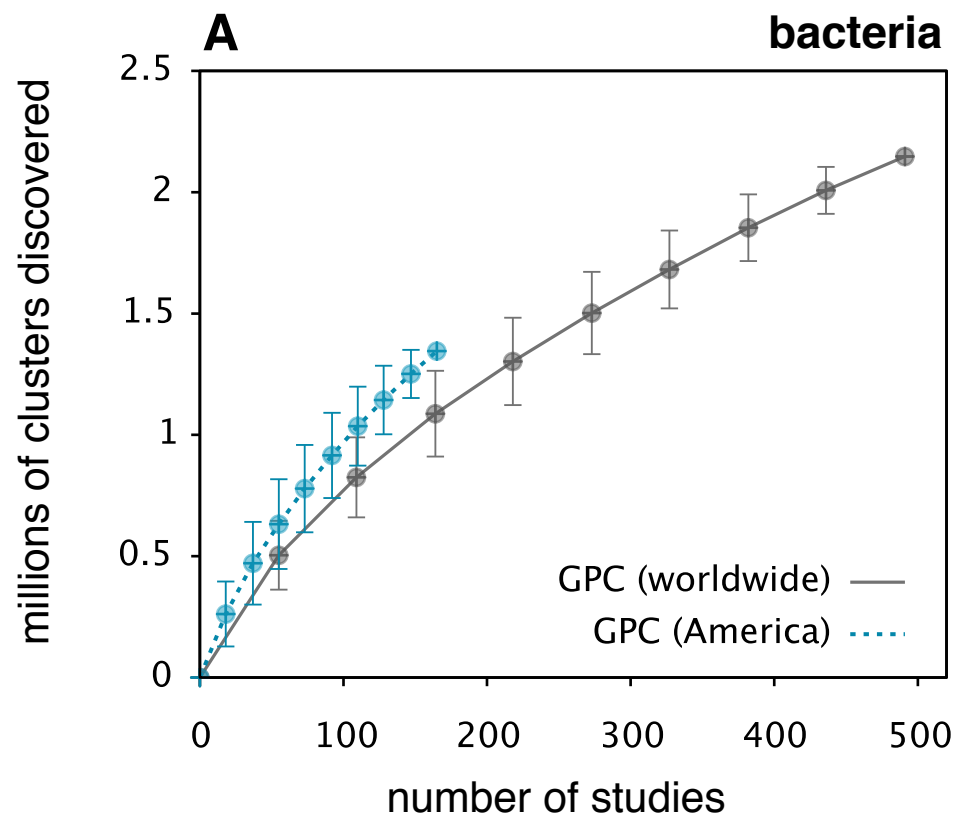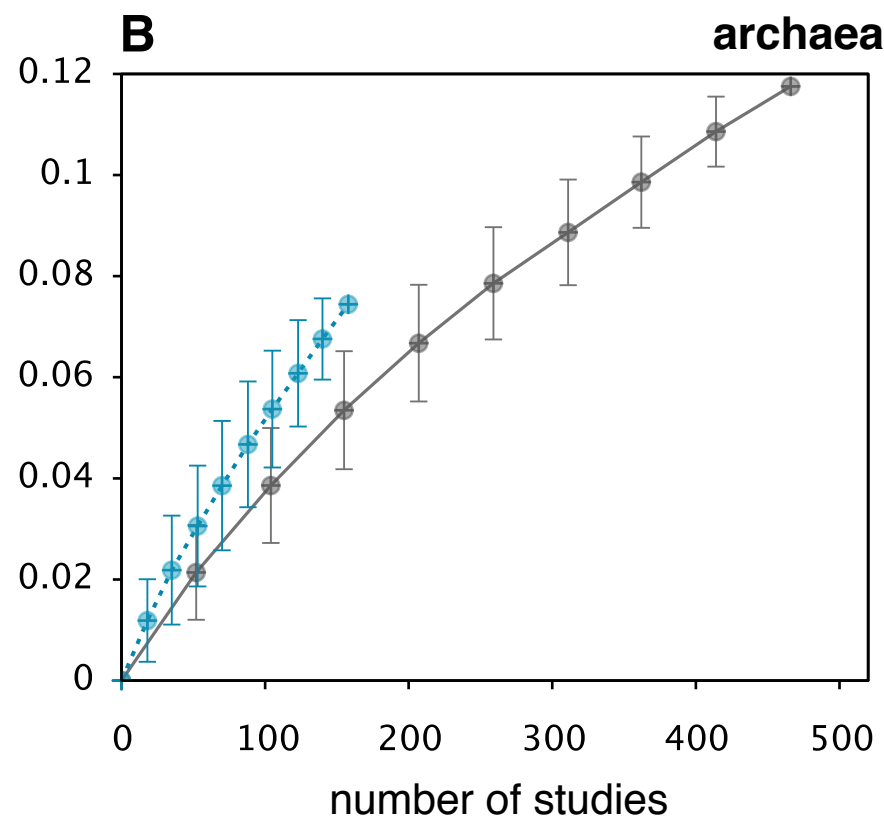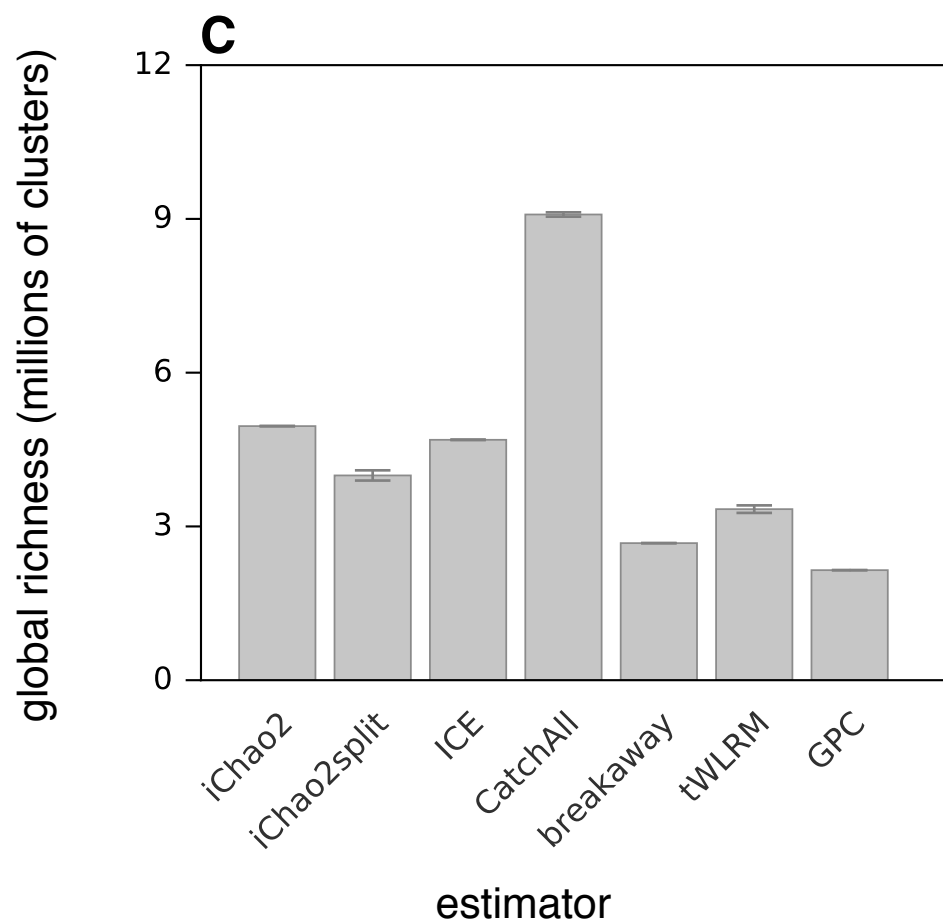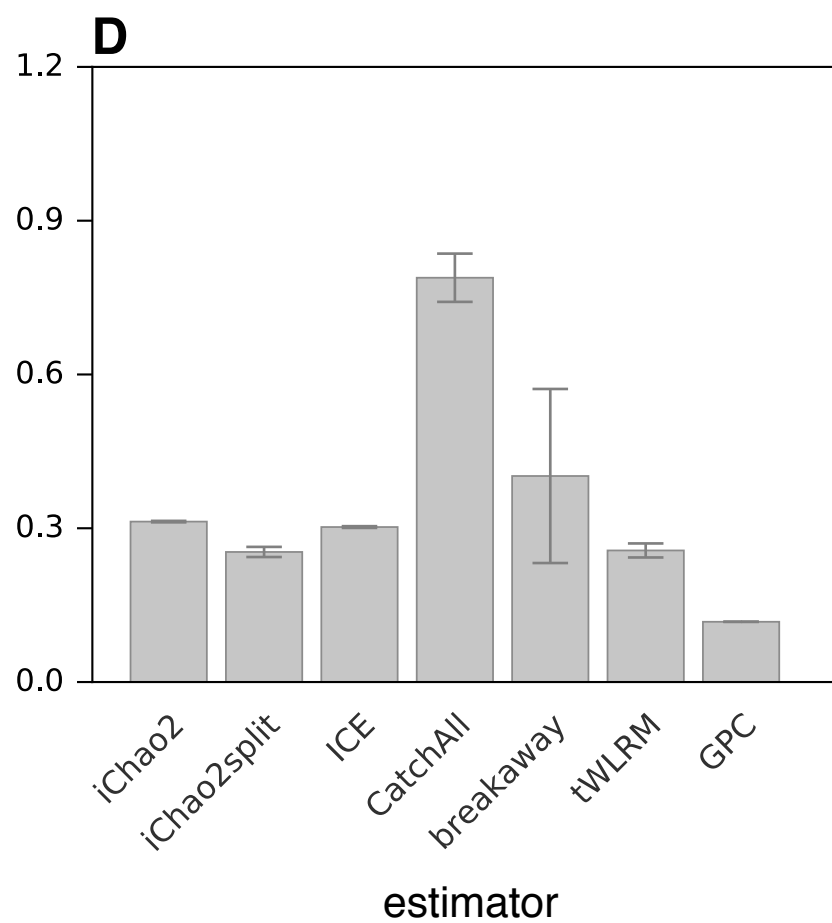

Supplement: S4 Fig — Accumulation curves, showing the number of bacterial (A) and archaeal (B) clusters (99% similarity in the 16S-V4 region) discovered, depending on the number of distinct studies included. Curves are averaged over 100 random subsamplings, and whiskers show corresponding standard deviations. Continuous curves were calculated using all studies (worldwide), while blue dashed curves were calculated using solely studies performed in the Americas or near American coasts. (PDF) [file pbio.3000106.s009.pdf]

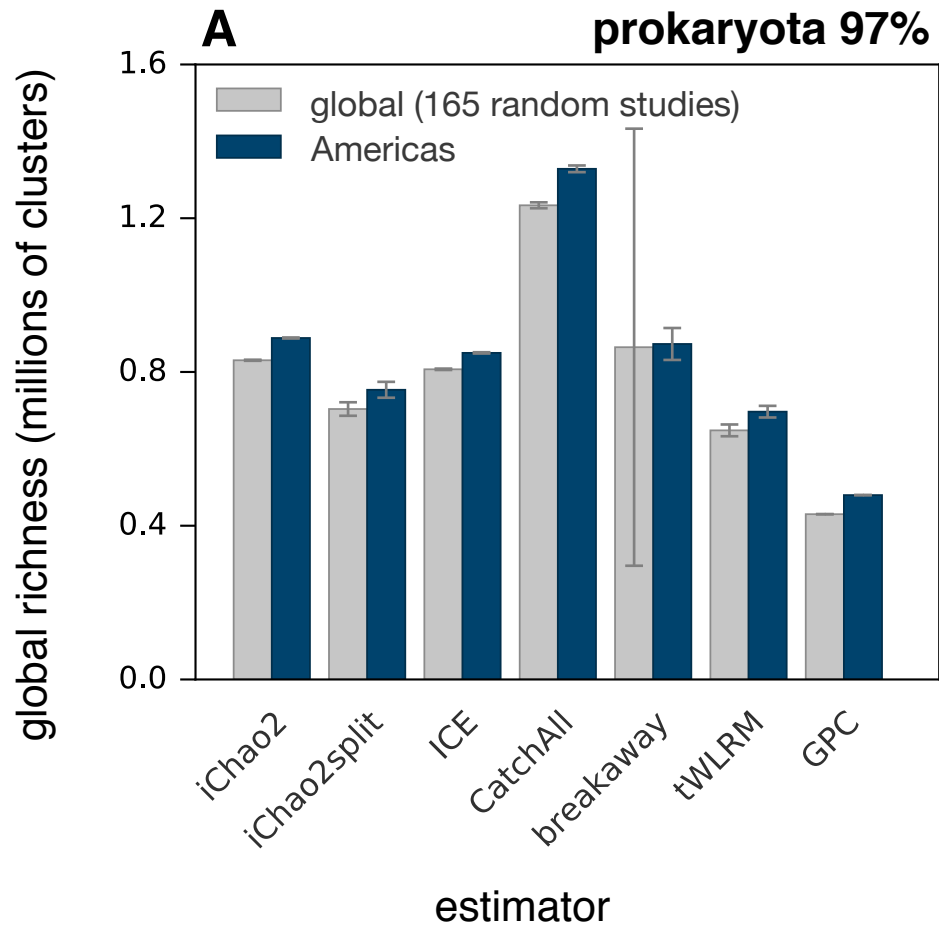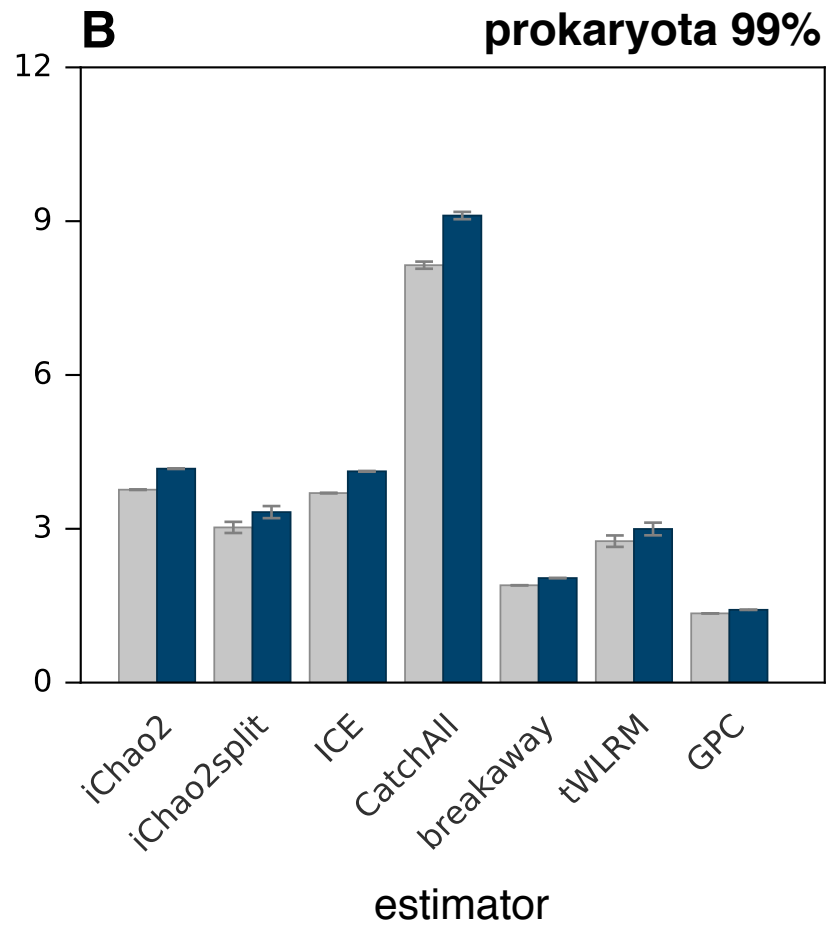

Supplement: S5 Fig — Prokaryotic 16S cluster richness at 97% (A) and 99% (B) clustering similarity, estimated using various statistical estimators and based on studies from the Americas (blue bars) or an equal number of studies chosen randomly from the global data set (grey bars). The number of OTUs discovered by the GPC is included for comparison (last bar). Error bars indicate standard errors, estimated from the underlying models; most standard errors are likely underestimated by the models, so the variability between models is probably a more honest assessment of uncertainty. GPC, Global Prokaryotic Census; OTU, operational taxonomic unit. (PDF) [file pbio.3000106.s010.pdf]

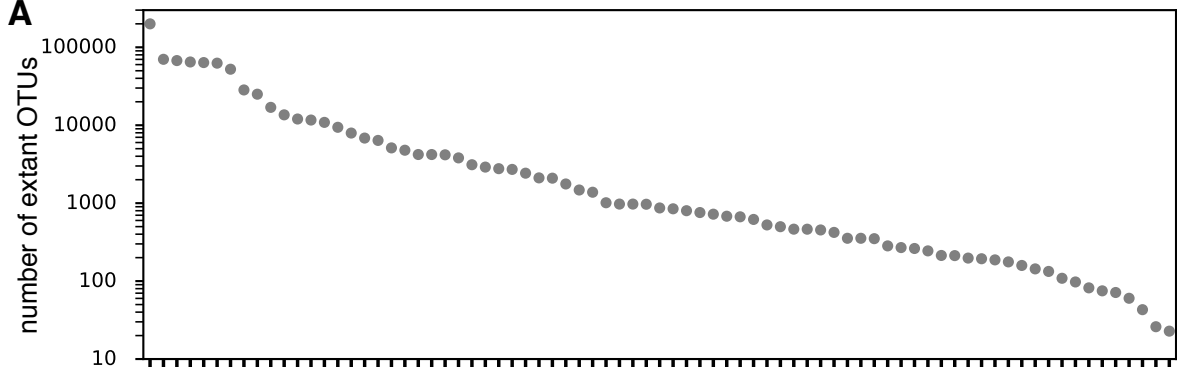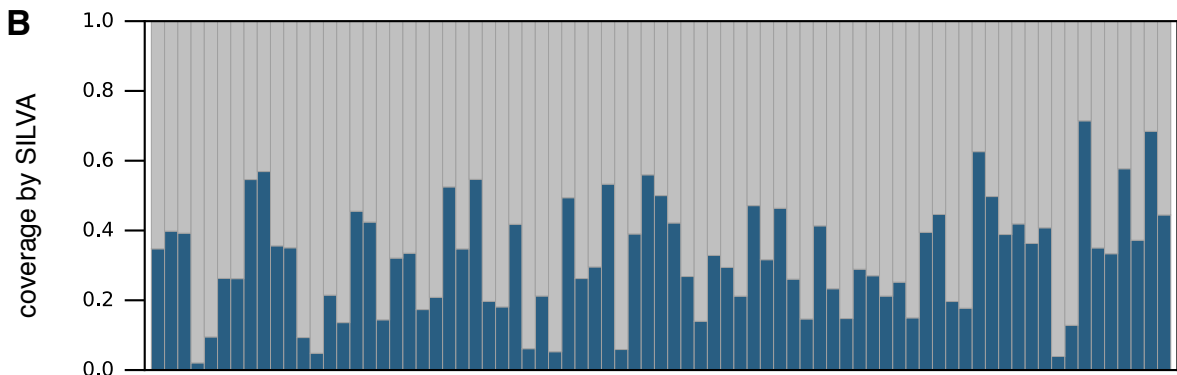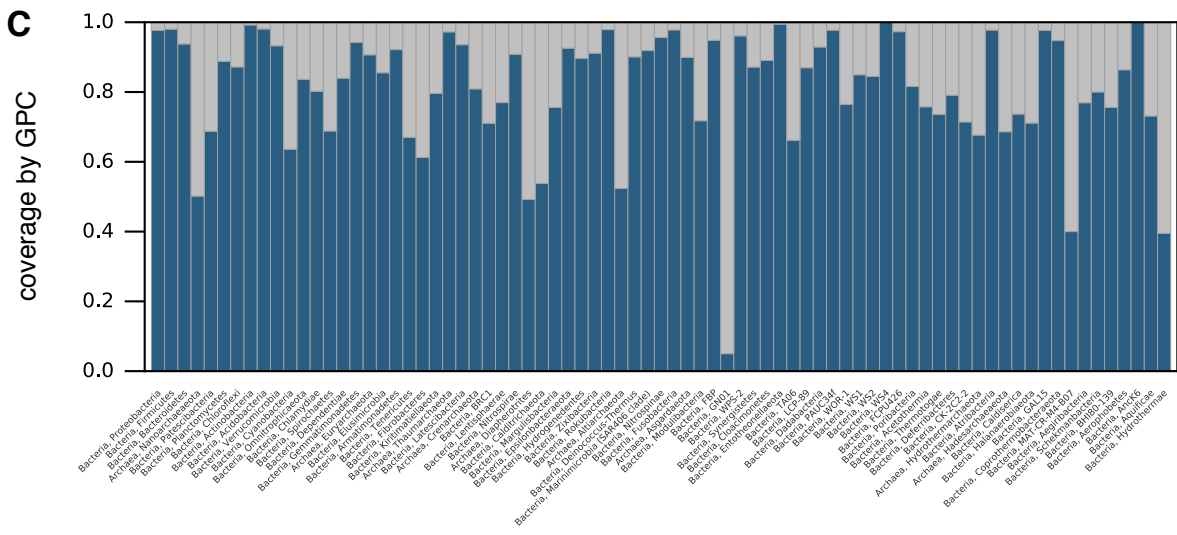

Supplement: S6 Fig — (A) Estimated number of OTUs (97% similarity in the 16S-V4 region) globally, within various prokaryotic phyla. Estimated based on the coverage of SILVA by the GPC (subfigure C) and the number of OTUs in the GPC. Only phyla including at least 10 entries in SILVA (release 132, set NR99) [14] and estimated to contain at least 10 extant OTUs are considered. (B) Fraction of GPC OTUs that could be mapped to SILVA NR99 at similarity ≥97%, as a proxy for global OTU richness covered by SILVA, within the same phyla as in A. (C) Fraction of SILVA NR99 sequences that could be mapped to the GPC at similarity ≥97%, as a proxy for global OTU richness covered by the GPC, within the same phyla as in A. GPC, Global Prokaryotic Census; NR, nonredundant; OTU, operational taxonomic unit; SILVA. (PDF) [file pbio.3000106.s011.pdf]

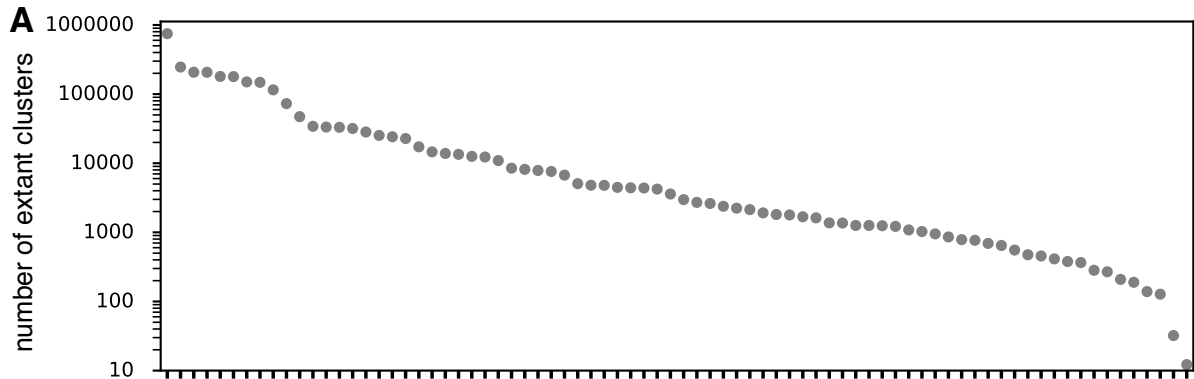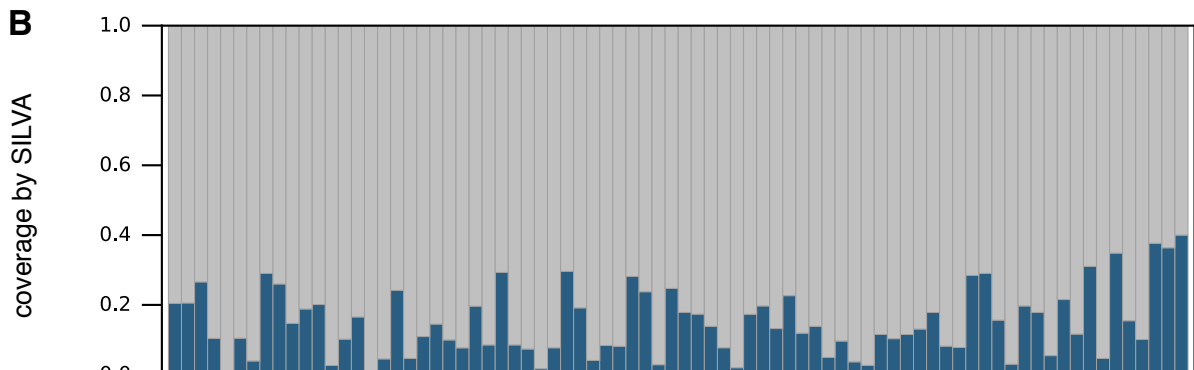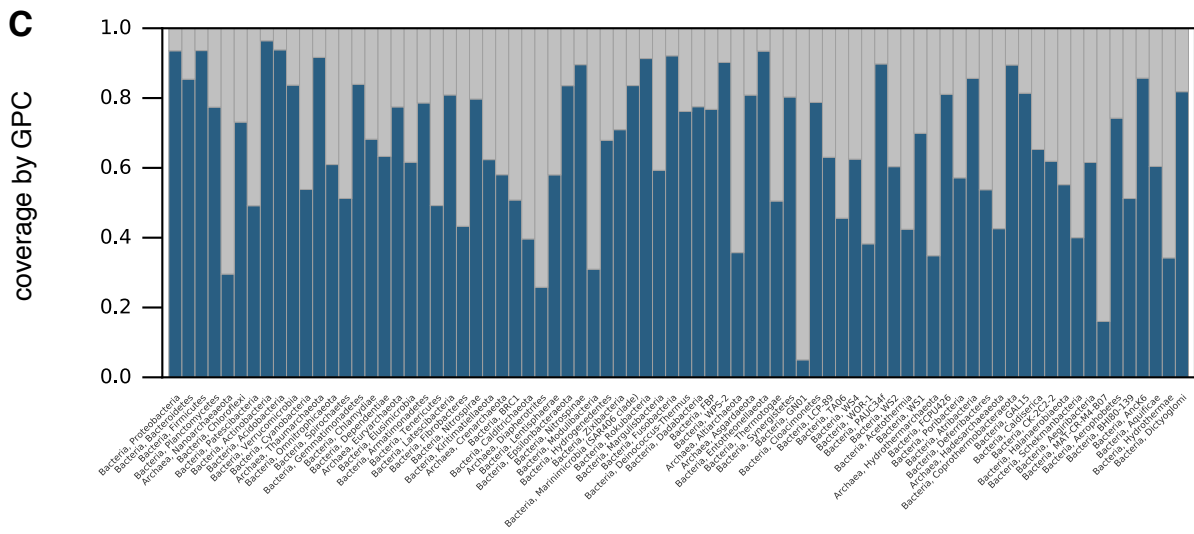

Supplement: S7 Fig — (A) Estimated number of 16S sequence clusters (99% similarity in the 16S-V4 region) globally, within various prokaryotic phyla. Estimated based on the coverage of SILVA by the GPC (subfigure C) and the number of clusters in the GPC. Only phyla including at least 10 entries in SILVA (release 132, set NR99) and estimated to contain at least 10 extant clusters are shown. (B) Fraction of GPC clusters that could be mapped to SILVA NR99 at similarity ≥99%, as a proxy for global OTU richness covered by SILVA, within the same phyla as in A. (C) Fraction of SILVA NR99 sequences that could be mapped to GPC clusters at similarity ≥99%, as a proxy for global cluster richness covered by the GPC, within the same phyla as in A. GPC, Global Prokaryotic Census; NR, nonredundant; OTU, operational taxonomic unit; SILVA. (PDF) [file pbio.3000106.s012.pdf]

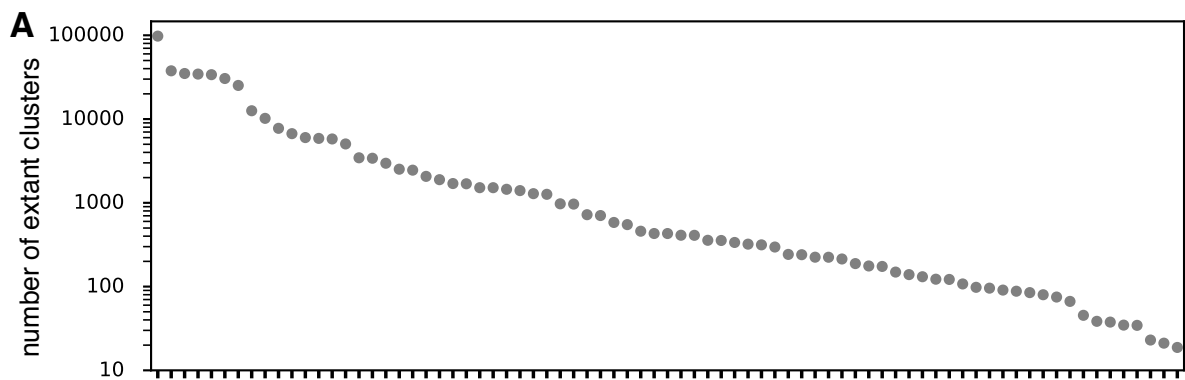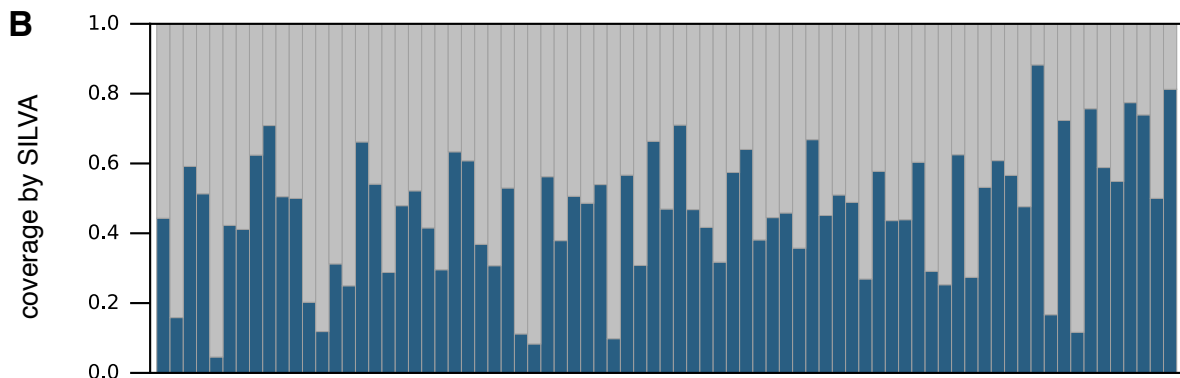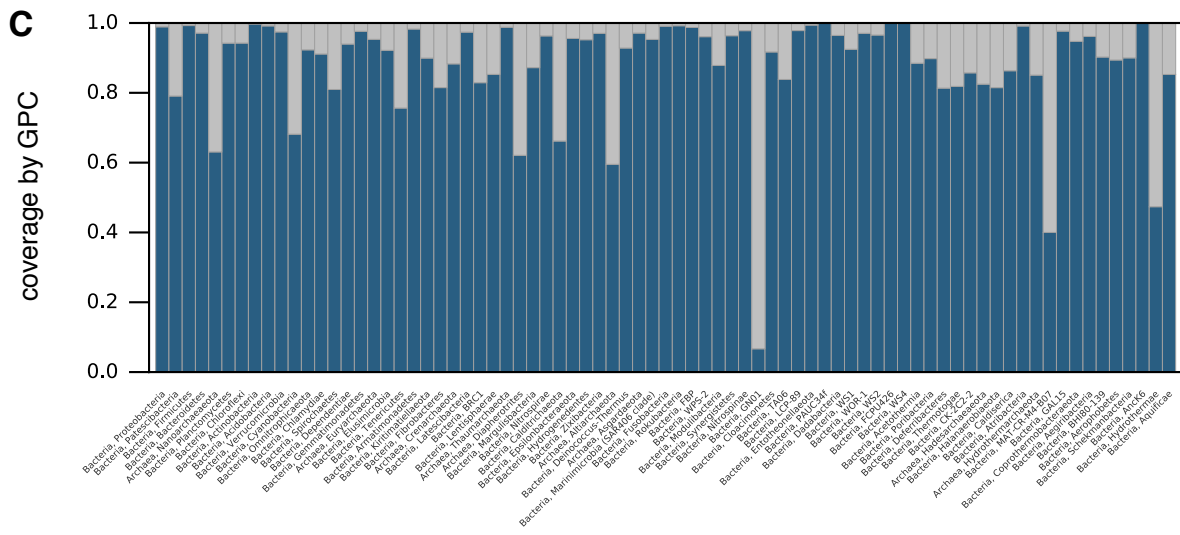

Supplement: S8 Fig — (A) Estimated number of 16S sequence clusters (95% similarity in the 16S-V4 region) globally, within various prokaryotic phyla. Estimated based on the coverage of SILVA by the GPC (subfigure C) and the number of clusters in the GPC. Only phyla including at least 10 entries in SILVA (release 132, set NR99) and estimated to contain at least 10 extant clusters are shown. (B) Fraction of GPC clusters that could be mapped to SILVA NR99 at similarity ≥95%, as a proxy for global cluster richness covered by SILVA, within the same phyla as in A. (C) Fraction of SILVA NR99 sequences that could be mapped to GPC clusters at similarity ≥95%, as a proxy for global cluster richness covered by the GPC, within the same phyla as in A. GPC, Global Prokaryotic Census; NR, nonredundant; SILVA. (PDF) [file pbio.3000106.s013.pdf]

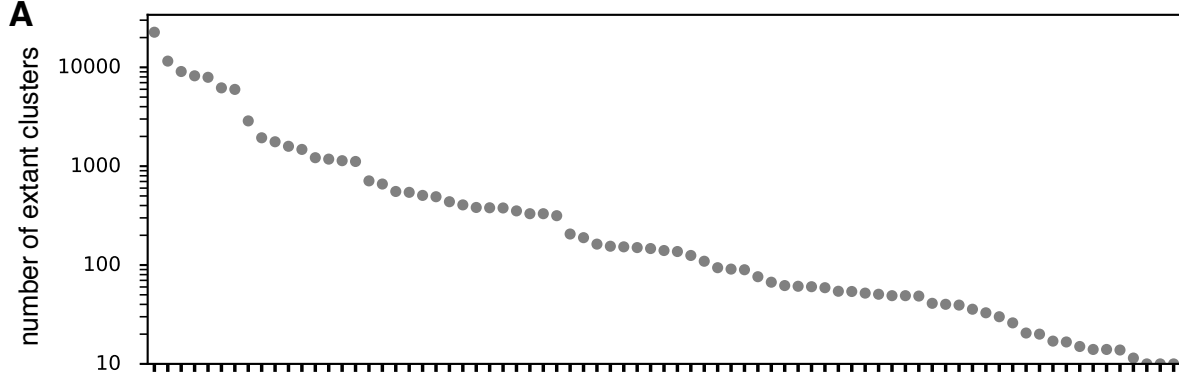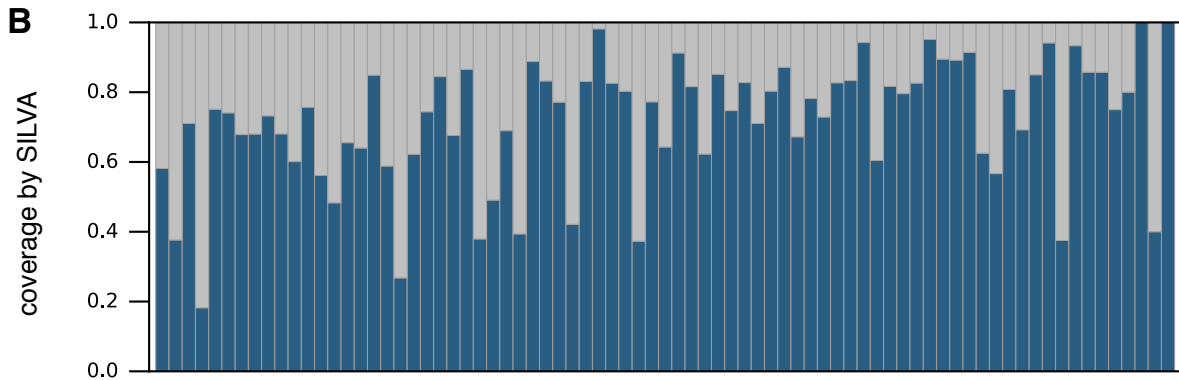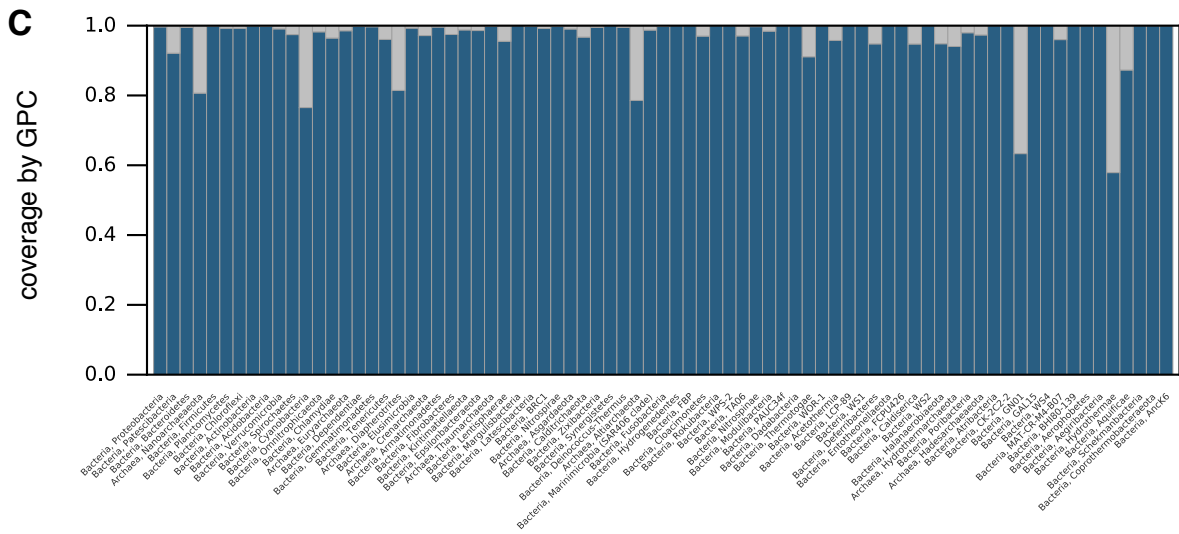

Supplement: S9 Fig — (A) Estimated number of 16S sequence clusters (90% similarity in the 16S-V4 region) globally, within various prokaryotic phyla. Estimated based on the coverage of SILVA by the GPC (subfigure C) and the number of clusters in the GPC. Only phyla including at least 10 entries in SILVA (release 132, set NR99) and estimated to contain at least 10 extant clusters are shown. (B) Fraction of GPC clusters that could be mapped to SILVA NR99 at similarity ≥90%, as a proxy for global cluster richness covered by SILVA, within the same phyla as in A. (C) Fraction of SILVA NR99 sequences that could be mapped to GPC clusters at similarity ≥90%, as a proxy for global cluster richness covered by the GPC, within the same phyla as in A. GPC, Global Prokaryotic Census; NR, nonredundant; SILVA. (PDF) [file pbio.3000106.s014.pdf]

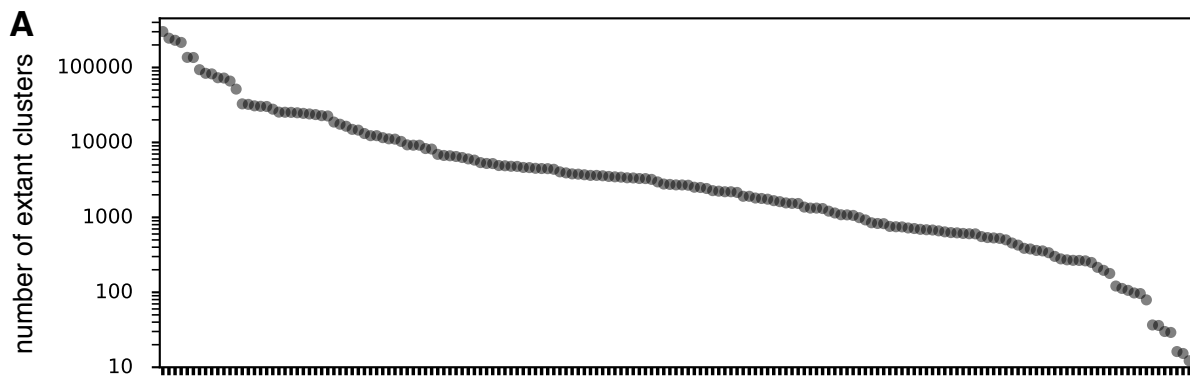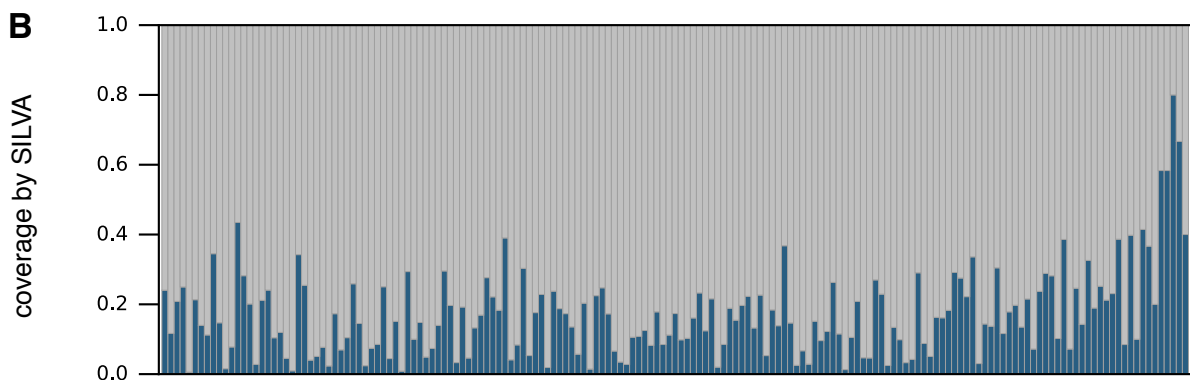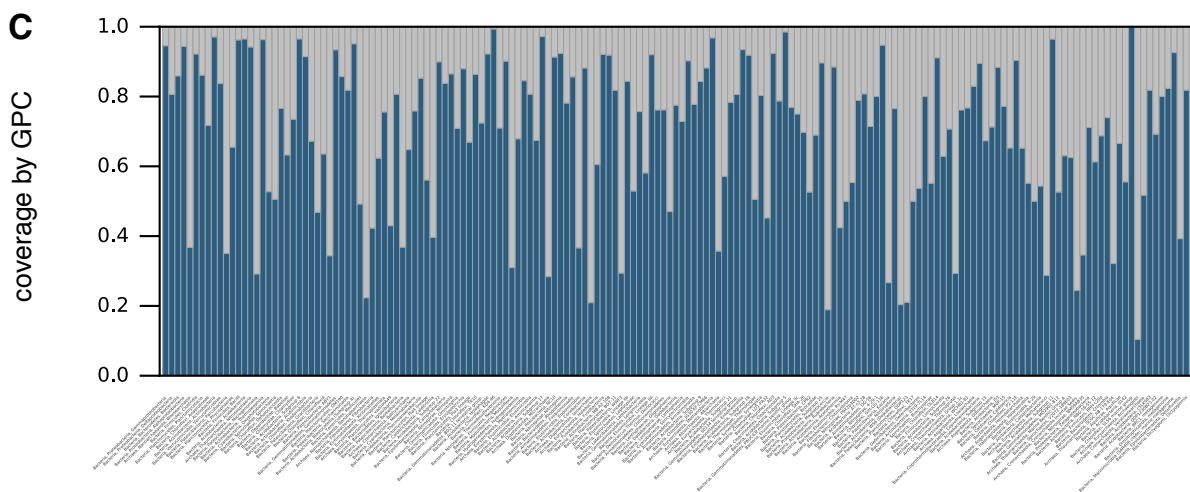

Supplement: S10 Fig — (A) Estimated number of 16S sequence clusters (99% similarity in the 16S-V4 region) globally, within various prokaryotic classes. Estimated based on the coverage of SILVA by the GPC (subfigure C) and the number of clusters in the GPC. Only classes including at least 10 entries in SILVA (release 132, set NR99) and estimated to contain at least 10 extant clusters are shown. (B) Fraction of GPC clusters that could be mapped to SILVA NR99 at similarity ≥99%, as a proxy for global cluster richness covered by SILVA, within the same classes as in A. (C) Fraction of SILVA NR99 sequences that could be mapped to GPC clusters at similarity ≥99%, as a proxy for global cluster richness covered by the GPC, within the same classes as in A. GPC, Global Prokaryotic Census; NR, nonredundant; SILVA. (PDF) [file pbio.3000106.s015.pdf]

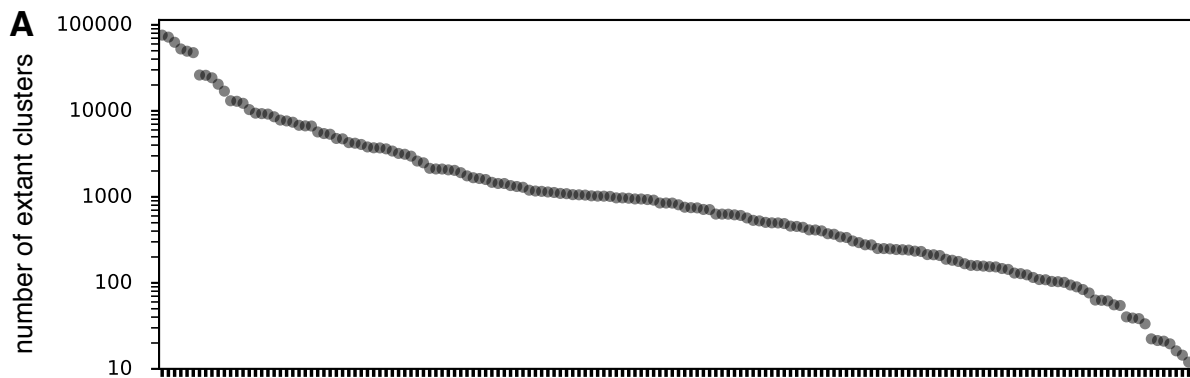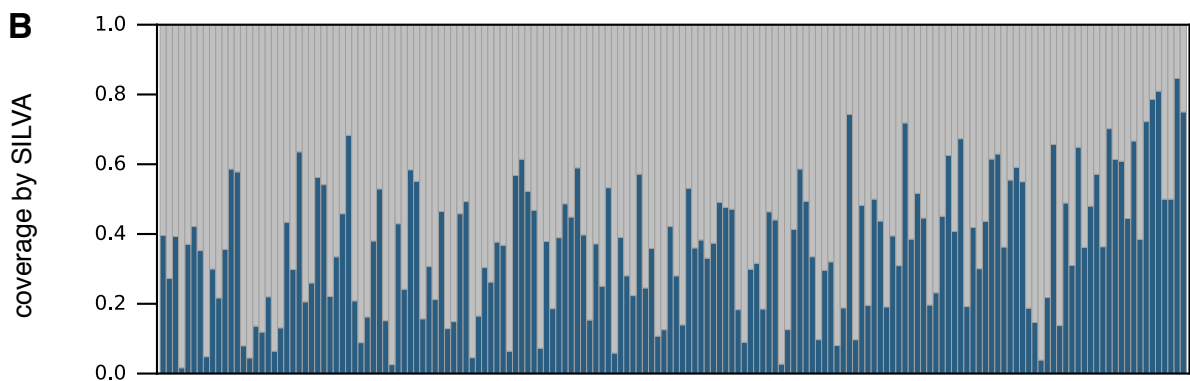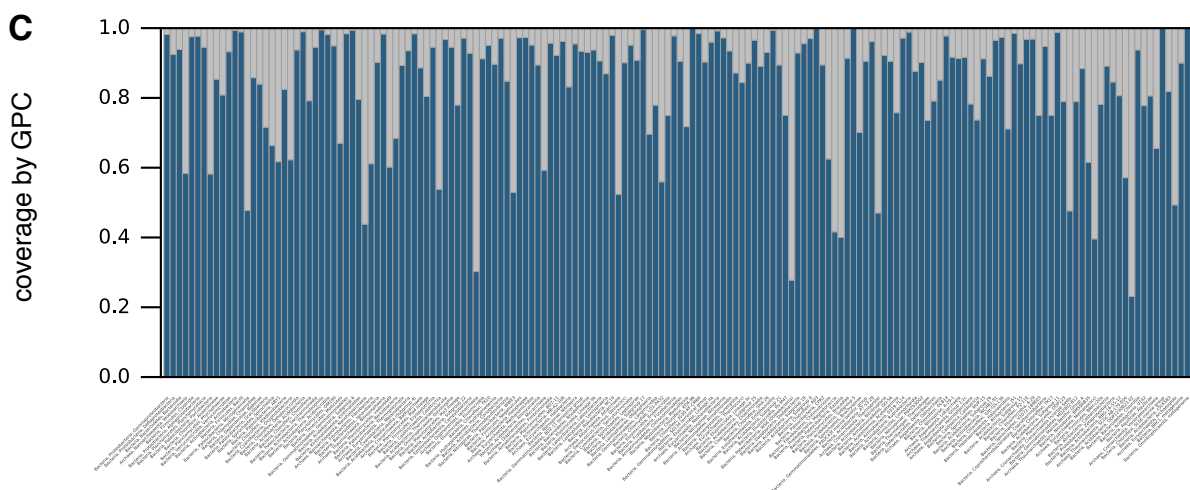

Supplement: S11 Fig — (A) Estimated number of 16S sequence clusters (97% similarity in the 16S-V4 region) globally, within various prokaryotic classes. Estimated based on the coverage of SILVA by the GPC (subfigure C) and the number of clusters in the GPC. Only classes including at least 10 entries in SILVA (release 132, set NR99) and estimated to contain at least 10 extant clusters are shown. (B) Fraction of GPC clusters that could be mapped to SILVA NR99 at similarity ≥97%, as a proxy for global OTU richness covered by SILVA, within the same classes as in A. (C) Fraction of SILVA NR99 sequences that could be mapped to GPC clusters at similarity ≥97%, as a proxy for global cluster richness covered by the GPC, within the same classes as in A. GPC, Global Prokaryotic Census; NR, nonredundant; OTU, operational taxonomic unit; SILVA. (PDF) [file pbio.3000106.s016.pdf]

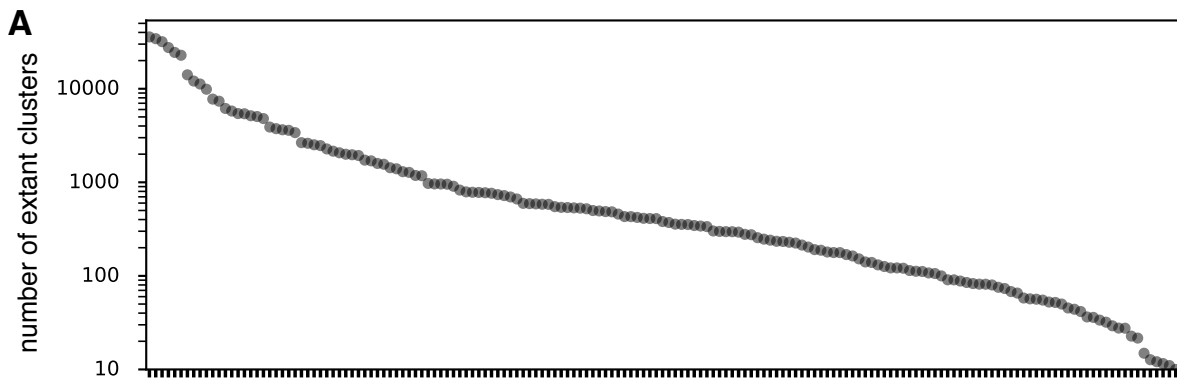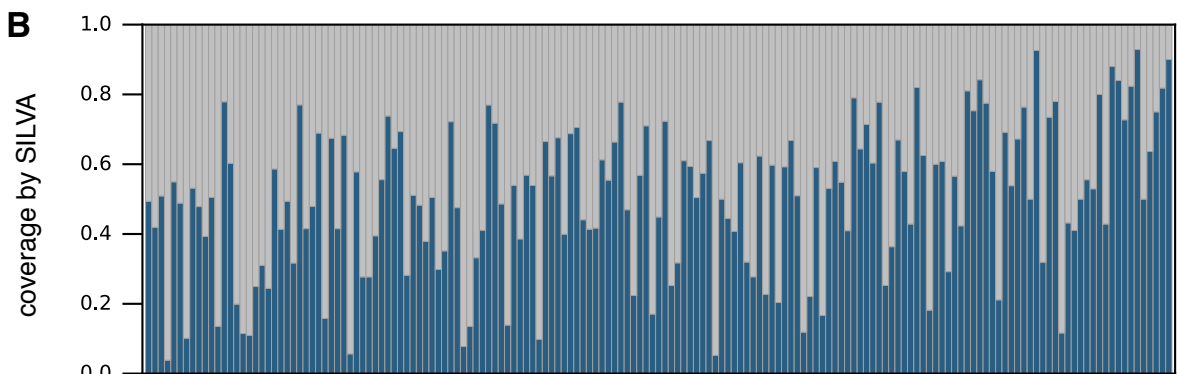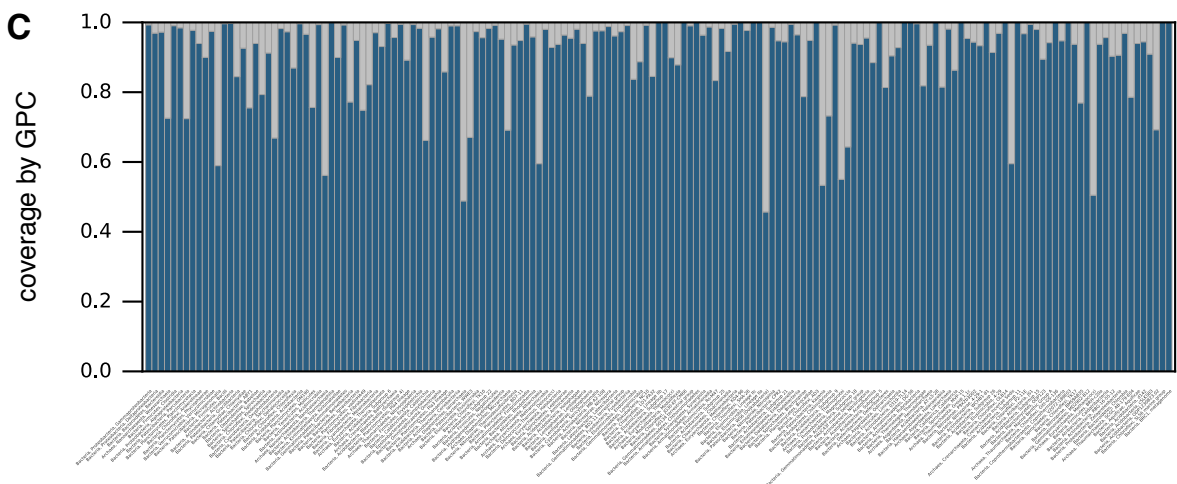

Supplement: S12 Fig — (A) Estimated number of 16S sequence clusters (95% similarity in the 16S-V4 region) globally, within various prokaryotic classes. Estimated based on the coverage of SILVA by the GPC (subfigure C) and the number of clusters in the GPC. Only classes including at least 10 entries in SILVA (release 132, set NR99) and estimated to contain at least 10 extant clusters are shown. (B) Fraction of GPC clusters that could be mapped to SILVA NR99 at similarity ≥95%, as a proxy for global cluster richness covered by SILVA, within the same classes as in A. (C) Fraction of SILVA NR99 sequences that could be mapped to GPC clusters at similarity ≥95%, as a proxy for global cluster richness covered by the GPC, within the same classes as in A. GPC, Global Prokaryotic Census; NR, nonredundant; SILVA. (PDF) [file pbio.3000106.s017.pdf]

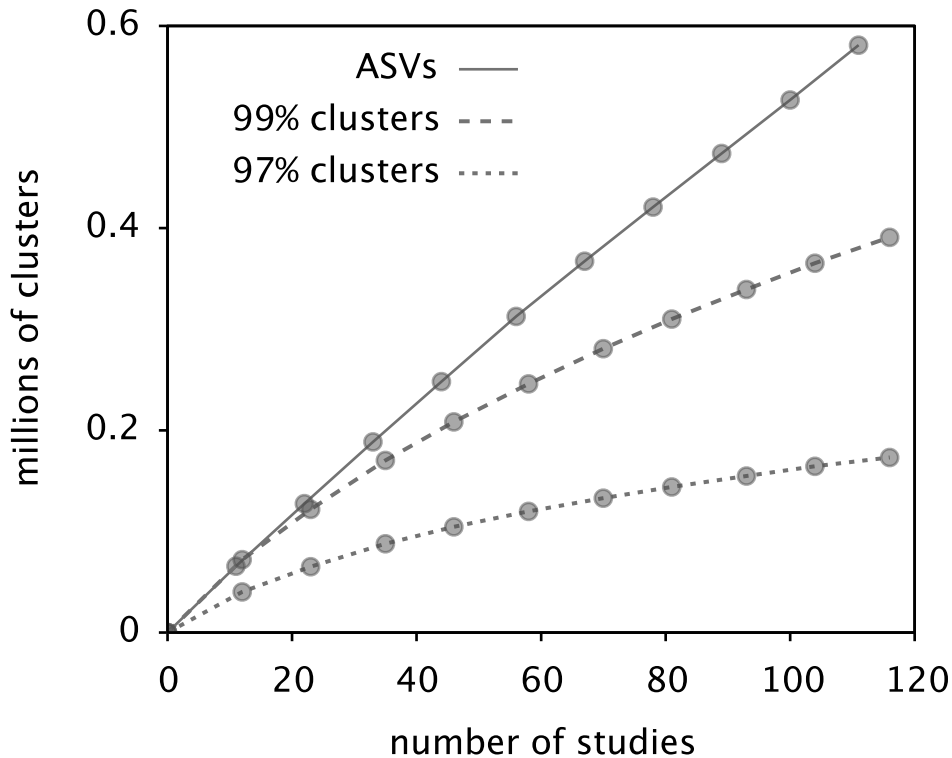

Supplement: S13 Fig — Number of prokaryotic 16S-V4 sequence clusters discovered at various similarity thresholds (97%, 99%, and 100%) and for various numbers of studies included. Only a subset of 111 studies were used in this analysis (subset "AG"). Clusters at 97% and 99% were generated using cd-hit-otu, as described in the main article; clusters at 100% correspond to exact ASVs, generated using DADA2. ASV, amplicon sequence variant; DADA2; GPC, Global Prokaryotic Census. (PDF) [file pbio.3000106.s018.pdf]

97%-clusters

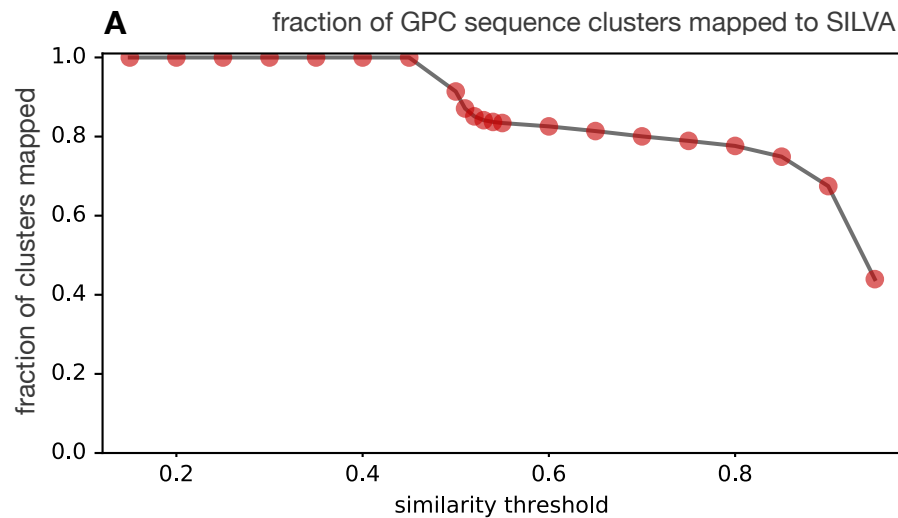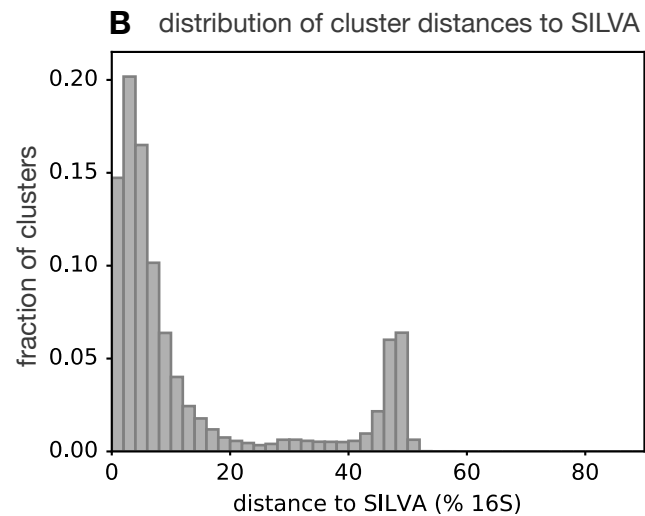

99%-clusters

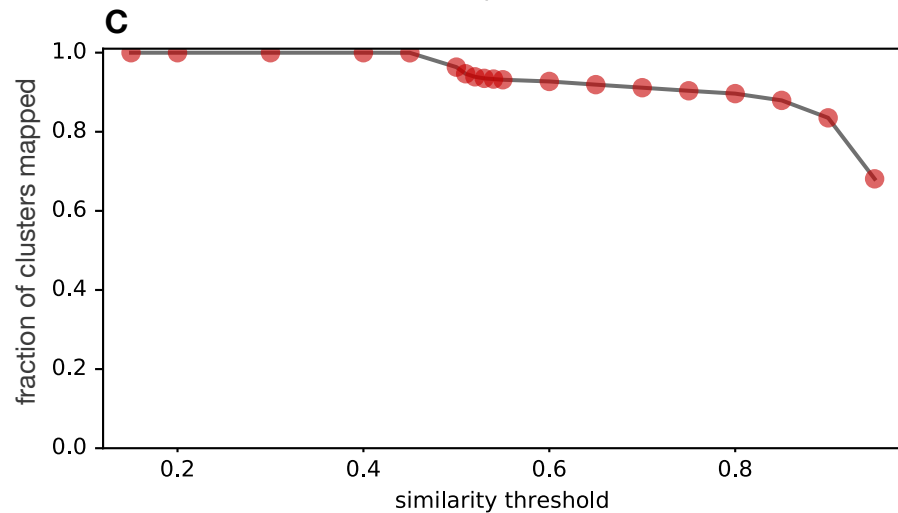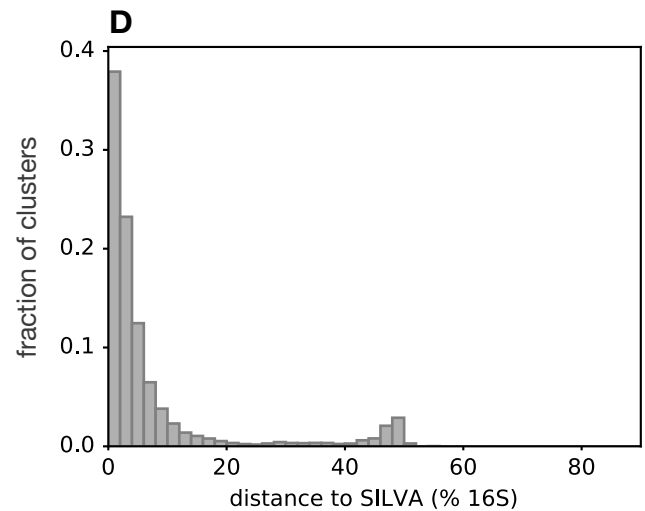

Supplement: S14 Fig — (A) Fraction of GPC 16S-V4 sequences clusters (clustered at 97% identity) that could be matched to a SILVA entry at various similarity thresholds (fraction of nucleotide mismatches). Note the jump at around 50% similarity. (B) Distribution of distances between GPC 16S-V4 sequence clusters (clustered at 97% identity) and SILVA (measured as percent of nucleotide mismatches to the closest match in SILVA). Note the sharp peak within the distance interval of 40%–50%. Figures A and B contain the same information, shown in alternative ways. Note that the horizontal axis shows similarities in A and distances in B. (C, D) Same as A and B but for GPC sequence clusters at 99% identity. Observe that the peak in the 40%–50% distance interval is much smaller for 99%-clusters than for 97%-clusters. Also see S15 Fig for a comparison with exact amplicon sequence variants. GPC, Global Prokaryotic Census; SILVA. (PDF) [file pbio.3000106.s019.pdf]

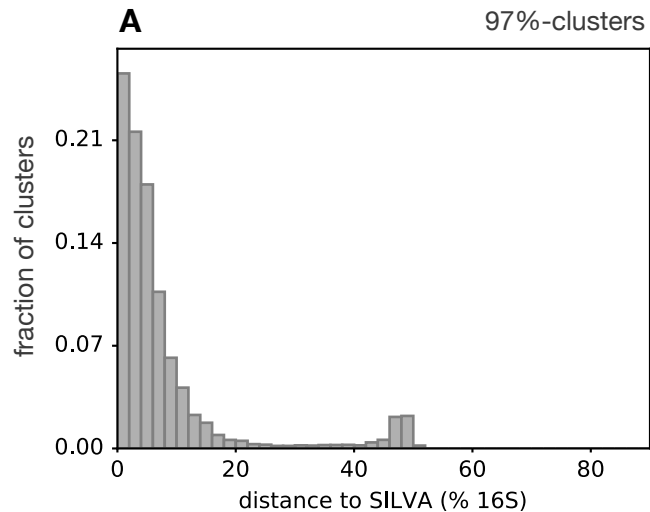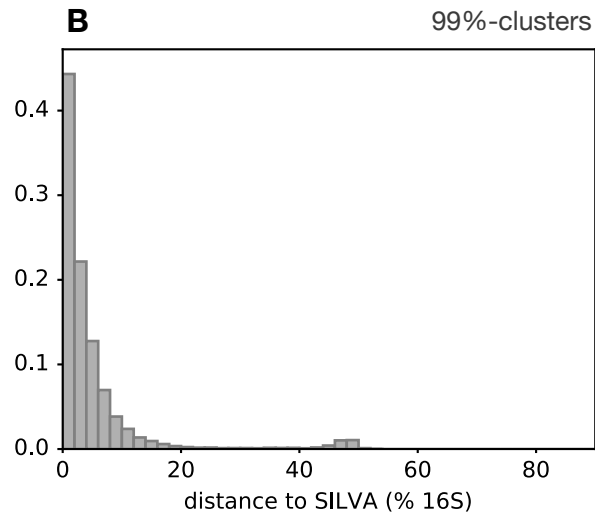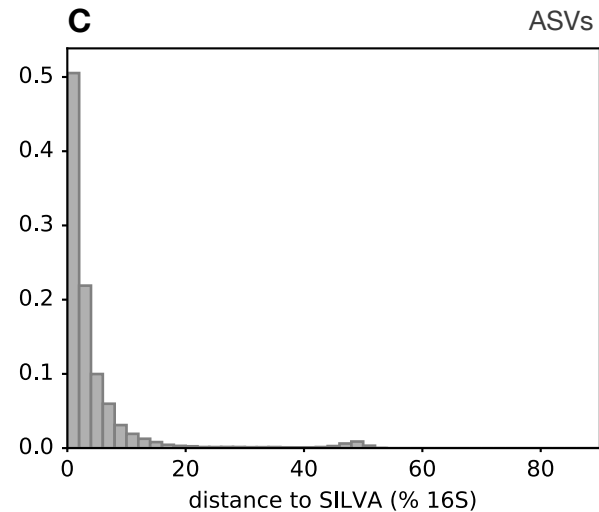

Supplement: S15 Fig — (A) Distribution of distances between 16S-V4 sequence clusters (clustered at 97% identity) and SILVA (measured as percent of nucleotide mismatches to the closest match in SILVA). Clusters were generated from a subset of 111 studies (subset "AG"). Note the peak within the distance interval 40%–50% and the absence of any OTUs to the right of that peak. (B) Similar to A but for sequence clusters at 99% identity. (C) Similar to A but for exact ASVs generated using DADA2. Observe that the peak in the 40%–50% distance interval is much smaller for 99%-clusters than for 97%-clusters and almost disappears in the case of ASVs. ASV, amplicon sequence variant; DADA2; GPC, Global Prokaryotic Census; OTU, operational taxonomic unit; SILVA. (PDF) [file pbio.3000106.s020.pdf]
