# Supplementary material for: A census-based estimate of Earth's bacterial and archaeal diversity
Source: PLoS Biol. 2019 Feb 4;17(2):e3000106. doi: 10.1371/journal.pbio.3000106 (PMC6361415; doi:10.1371/journal.pbio.3000106)
Supplement: S1 Table — Number of extant prokaryotic 16S sequence clusters (at 90%, 95%, 97%, or 99% similarities in the 16S-V4 region), estimated using various methods, including iChao2, iChao2split, ICE, CatchAll, breakaway, tWLRM, based on the coverage of SILVA, or the RDP (see main text for details), and based on a log-normal model fitted to OTU MRAs. Uncertainties (±) correspond to standard errors, wherever applicable. The last row lists the number of clusters discovered by the GPC. NA indicates that the estimator did not converge. GPC, Global Prokaryotic Census; ICE, incidence coverage-based estimator; MRA, mean relative abundance; NA, not available; OTU, operational taxonomic unit; RDP, Ribosomal Database Project; SILVA; tWLRM, transformed weighted linear regression model. (PDF) [file pbio.3000106.s021.pdf]

Table S1: **Estimated numbers of extant prokaryotic 16S clusters worldwide**.

**estimator**	**90%**		**95%**		**97%**		**99%**	
iChao2	145,226	±330	572,163	±672	1,148,742	±1,064	5,267,919	±5016
iChao2split	130,023	±1,111	508,755	±7,098	1,012,948	±19,228	4,252,231	±82,077
ICE	134,350	±329	538,437	±810	1,094,340	±1,380	4,991,376	±5,535
CatchAll	163,531	±745	683,457	±1,980	1,441,048	±3,664	9,686,211	±44,323
breakaway	211,981	±56,712	782,987	±86,891	1,588,567	±204,133	2,835,478	±1,111
tWLRM	117,022	±632	457,274	±3,436	901,901	±8,157	3,548,723	±78,949
b.o. SILVA	107,650		403,585		771,234		2,513,950	
b.o. RDP	110,146		426,381		832,420		2,825,940	
MRA	119,991		451,044		886,291		2,462,942	
GPC	107,215		395,598		739,880		2,264,506	
