# Supplementary material for: A census-based estimate of Earth's bacterial and archaeal diversity
Source: PLoS Biol. 2019 Feb 4;17(2):e3000106. doi: 10.1371/journal.pbio.3000106 (PMC6361415; doi:10.1371/journal.pbio.3000106)
Supplement: S2 Table — Fraction of recaptured (at 97% similarity) prokaryotic 16S sequences in third party data sets, including the EMP, the SILVA (NR99) database release 132, 16S sequences assembled from metagenomes (UBA), bacterial 16S sequences extracted from IMG/M metagenomes, the RDP release 11, and the Genome Taxonomic Database release 86.1, by GPC OTUs. In cases in which the number of OTUs in the third party data set was low (<1,000), the numbers of OTUs compared are indicated in brackets. EMP, Earth Microbiome Project; GPC, Global Prokaryotic Census; IMG/M, Integrated Microbial Genomes and Microbiomes; NR, nonredundant; OTU, operational taxonomic unit; RDP, Ribosomal Database Project; SILVA; UBA, Uncultivated Bacteria and Archaea. (PDF) [file pbio.3000106.s022.pdf]

Table S2: **Recapture fractions of other datasets by the GPC (at 97% similarity).**

taxon	EMP	SILVA	UBA	IMG/M	RDP	GTDB
Bacteria	0.93	0.96	0.90 (393/436)	0.93	0.89	0.94
Archaea	0.81	0.88	0.92 (138/150)	NA	0.90	0.78
Cyanobacteria	0.90	0.95	1.00 (5/5)	0.96	0.88	0.97
Proteobacteria	0.94	0.98	0.98 (117/120)	0.97	0.91	0.98
Firmicutes	0.88	0.98	0.86 (25/29)	0.97	0.95	0.99
