# Supplementary material for: A census-based estimate of Earth's bacterial and archaeal diversity
Source: PLoS Biol. 2019 Feb 4;17(2):e3000106. doi: 10.1371/journal.pbio.3000106 (PMC6361415; doi:10.1371/journal.pbio.3000106)
Supplement: S3 Table — Fraction of recaptured (at ≥99% similarity) prokaryotic 16S sequences in third party data sets, including the EMP, the SILVA (NR99) database release 132, 16S sequences assembled from metagenomes (UBA), bacterial 16S sequences extracted from IMG/M metagenomes, and the RDP release 11, by 16S sequence clusters (99% similarity) in the GPC. EMP, Earth Microbiome Project; GPC, Global Prokaryotic Census; IMG/M, Integrated Microbial Genomes and Microbiomes; NR, nonredundant; RDP, Ribosomal Database Project; SILVA; UBA, Uncultivated Bacteria and Archaea. (PDF) [file pbio.3000106.s023.pdf]

Table S3: **Recapture fractions of other datasets by the GPC (at 99% similarity)**.

taxon	EMP	SILVA	UBA	IMG/M	RDP	GTDB
Bacteria	0.82	0.91	0.80	0.88	0.80	0.90
Archaea	0.62	0.74	0.86	NA	0.79	0.66
