# Supplementary material for: A census-based estimate of Earth's bacterial and archaeal diversity
Source: PLoS Biol. 2019 Feb 4;17(2):e3000106. doi: 10.1371/journal.pbio.3000106 (PMC6361415; doi:10.1371/journal.pbio.3000106)
Supplement: S5 Table — Fraction of recaptured (at ≥90% similarity) prokaryotic 16S sequences in third party data sets, including the EMP, the SILVA (NR99) database release 132, 16S sequences assembled from metagenomes (UBA), bacterial 16S sequences extracted from IMG/M metagenomes, and the RDP release 11, by 16S sequence clusters (90% similarity) in the GPC. EMP, Earth Microbiome Project; GPC, Global Prokaryotic Census; IMG/M, Integrated Microbial Genomes and Microbiomes; NR, nonredundant; RDP, Ribosomal Database Project; SILVA; UBA, Uncultivated Bacteria and Archaea. (PDF) [file pbio.3000106.s025.pdf]

Table S5: **Recapture fractions of other datasets by the GPC (at 90% similarity)**.

**taxon**	**EMP**	**SILVA**	**UBA**	**IMG/M**	**RDP**	**GTDB**
Bacteria	0.98	0.997	0.98	0.98	0.97	0.99
Archaea	0.97	0.977	0.95	NA	0.97	0.92
