# Supplementary material for: A census-based estimate of Earth's bacterial and archaeal diversity
Source: PLoS Biol. 2019 Feb 4;17(2):e3000106. doi: 10.1371/journal.pbio.3000106 (PMC6361415; doi:10.1371/journal.pbio.3000106)
Supplement: S6 Table — Number of 16S clusters in SILVA release 132 within various taxa, obtained when clustering only the V4 region (200 bp, starting at E. coli position 516) or the full 16S gene (approximately 1,500 bp) at 97% or 99% similarity. SILVA. (PDF) [file pbio.3000106.s026.pdf]

Table S6: **Prokaryotic diversity contained in SILVA**.

taxon	V4 (97%)	full-length (97%)	V4 (99%)	full-length (99%)
Bacteria	94,552	256,483	216,405	603,135
Archaea	5,742	10,934	13,990	25,759
Cyanobacteria	1,641	2,739	3,150	7,873
Proteobacteria	26,718	77,338	66,514	239,834
Firmicutes	22,050	75,288	57,995	157,959
