# Supplementary material for: A census-based estimate of Earth's bacterial and archaeal diversity
Source: PLoS Biol. 2019 Feb 4;17(2):e3000106. doi: 10.1371/journal.pbio.3000106 (PMC6361415; doi:10.1371/journal.pbio.3000106)
Supplement: S7 Table — Number of 16S sequence clusters in the GPC with exactly two reads (N2) and probability that a single additional amplicon sequence would hit a GPC cluster (P, estimated using the Good–Turing frequency formula, see Methods for details) for various clustering similarities. GPC, Global Prokaryotic Census. (PDF) [file pbio.3000106.s027.pdf]

Table S7: **Estimated numbers of living prokaryotic cells represented by the GPC (at 90%, 95%, 97% or 99% similarity)**.

**taxon**	N_2	**P**
99% similarity	514,347	0.99881
97% similarity	87,940	0.99979
95% similarity	40,347	0.99991
90% similarity	8,274	0.99998
