# Supplementary material for: A census-based estimate of Earth's bacterial and archaeal diversity
Source: PLoS Biol. 2019 Feb 4;17(2):e3000106. doi: 10.1371/journal.pbio.3000106 (PMC6361415; doi:10.1371/journal.pbio.3000106)
Supplement: S3 Fig — Frequency histogram of MRAs of prokaryotic 16S clusters (A: 99% similarity, B: 95% similarity, C: 90% similarity) discovered by the GPC (grey continuous line), of clusters discovered by the rGPC (grey dashed lines), and of all extant clusters as estimated using a probabilistic model of OTU discovery (blue continuous curve). The probabilistic model was fitted separately for each MRA interval by comparing the discovery rates of the GPC and the rGPC. The blue dashed curve shows a log-normal distribution model fitted to the estimated MRA distribution of extant clusters (R2 > 0.96 in all cases). GPC, Global Prokaryotic Census; MRA, mean relative abundance; OTU, operational taxonomic unit; rGPC, rarefied variant of the GPC. (PDF) [file pbio.3000106.s008.pdf]

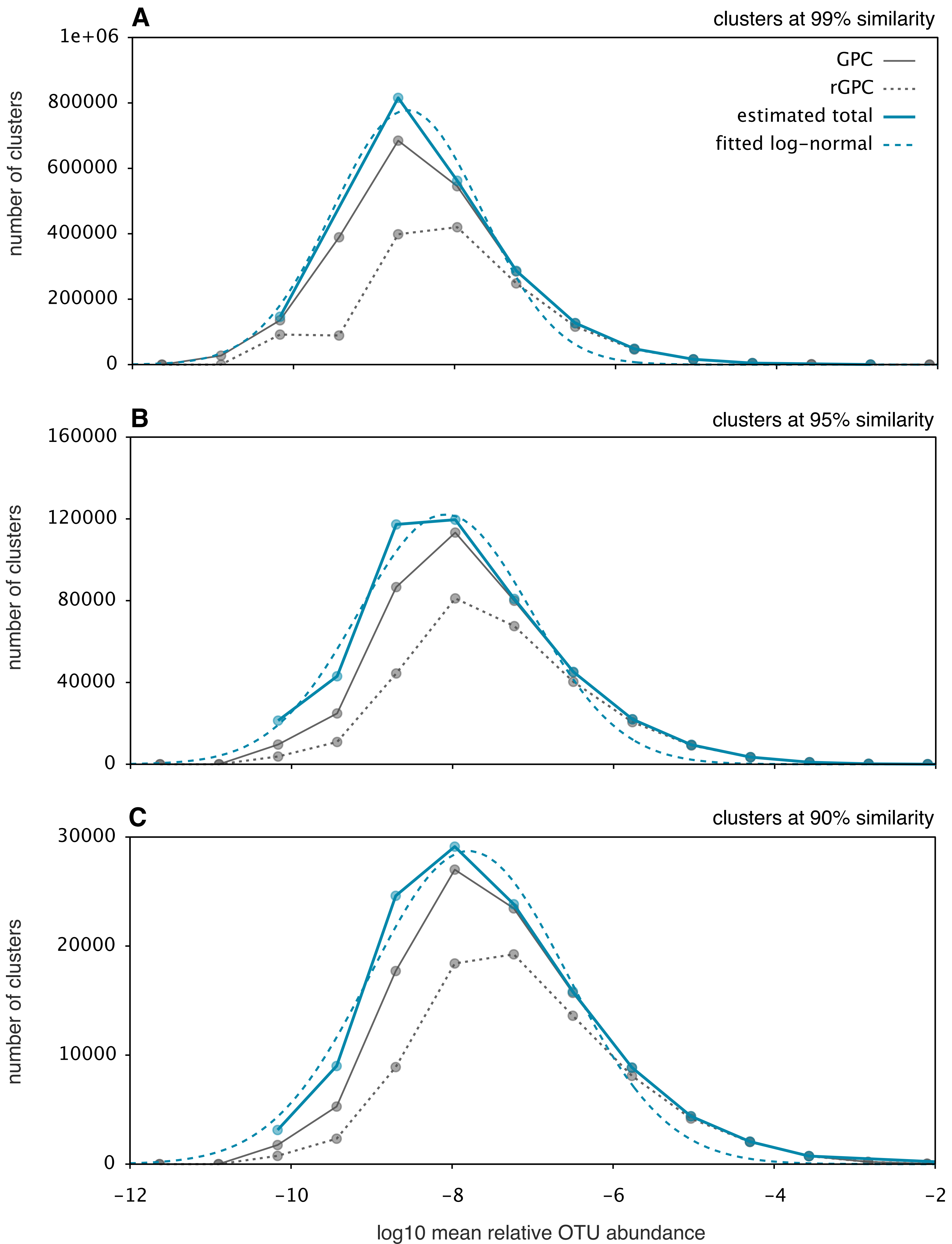

A
clusters at 99% similarity
GPC
rGPC
estimated total
fitted log-normal
1e+06
800000
600000
400000
200000
0
number of clusters
B
clusters at 95% similarity
160000
120000
80000
40000
0
number of clusters
C
clusters at 90% similarity
30000
20000
10000
0
number of clusters
−12
−10
−8
−6
−4
−2
log10 mean relative OTU abundance
